# Enhanced *in planta* Fitness through Adaptive Mutations in EfpR, a Dual Regulator of Virulence and Metabolic Functions in the Plant Pathogen *Ralstonia solanacearum*

**DOI:** 10.1371/journal.ppat.1006044

**Published:** 2016-12-02

**Authors:** Anthony Perrier, Rémi Peyraud, David Rengel, Xavier Barlet, Emmanuel Lucasson, Jérôme Gouzy, Nemo Peeters, Stéphane Genin, Alice Guidot

**Affiliations:** LIPM, Université de Toulouse, INRA, CNRS, Castanet-Tolosan, France; University of Toronto, CANADA

## Abstract

Experimental evolution of the plant pathogen *Ralstonia solanacearum*, where bacteria were maintained on plant lineages for more than 300 generations, revealed that several independent single mutations in the *efpR* gene from populations propagated on beans were associated with fitness gain on bean. In the present work, novel allelic *efpR* variants were isolated from populations propagated on other plant species, thus suggesting that mutations in *efpR* were not solely associated to a fitness gain on bean, but also on additional hosts. A transcriptomic profiling and phenotypic characterization of the *efpR* deleted mutant showed that EfpR acts as a global catabolic repressor, directly or indirectly down-regulating the expression of multiple metabolic pathways. EfpR also controls virulence traits such as exopolysaccharide production, swimming and twitching motilities and deletion of *efpR* leads to reduced virulence on tomato plants after soil drenching inoculation. We studied the impact of the single mutations that occurred in *efpR* during experimental evolution and found that these allelic mutants displayed phenotypic characteristics similar to the deletion mutant, although not behaving as complete loss-of-function mutants. These adaptive mutations therefore strongly affected the function of *efpR*, leading to an expanded metabolic versatility that should benefit to the evolved clones. Altogether, these results indicated that EfpR is a novel central player of the *R*. *solanacearum* virulence regulatory network. Independent mutations therefore appeared during experimental evolution in the evolved clones, on a crucial node of this network, to favor adaptation to host vascular tissues through regulatory and metabolic rewiring.

## Author Summary

Among plant pathogens of major economic and food crops, *Ralstonia solanacearum*, the causal agent of bacterial wilt, is recognized as one of the most destructive plant bacterial diseases. In addition, the emergence of new pathotypes, more aggressive and adapted to new hosts, has been reported. During an evolution experiment of *R*. *solanacearum*, where bacteria were maintained on plant lineages for more than 300 generations, we demonstrated that several single mutations in the regulatory gene *efpR* were associated with fitness gain on plants. However, the function of the EfpR regulator was totally unknown. In this work, we provided evidence that EfpR controls several metabolic pathways and important virulence traits of *R*. *solanacearum*. We then demonstrated that the single mutations selected in the *efpR* gene during the evolution experiment strongly alter the *efpR* expression, and thus enlarge the metabolic capacities of the bacterial cell. Altogether, our study reveals that EfpR is a novel key component of the complex regulatory network of the *R*. *solanacearum* cell, tightly linking the bacterial metabolism to virulence in response to multiple environmental signals.

## Introduction

Bacterial plant pathogens constitute a major threat to crop production. In addition, disease emergence can occur through rapid adaptation of many pathogens to new hosts [1,2]. Understanding how pathogens are adapting to new hosts is crucial for unraveling the mechanisms that drive disease emergence. One way to study evolution of pathogen adaptation to new hosts is to conduct experimental evolution of the pathogen in a given host over hundreds of generations [3–6]. The combination of experimental evolution with whole-genome sequencing has enabled the characterization of the mutations underlying the within-host fitness gain in various host-pathogen systems [4,6].

In a previous work, aiming at investigating the genetic bases of host adaptation in the bacterial plant pathogen *Ralstonia solanacearum*, we performed an experimental evolution of strain GMI1000 in different host plants [6]. *R*. *solanacearum* is the causal agent of bacterial wilt disease. It is recognized as one of the most destructive bacterial plant diseases affecting an unusually large host range of more than 250 plant species around the world, mainly in warm tropical climates [7,8]. *R*. *solanacearum* is a soilborne pathogen that infects the plants through the roots, invades the xylem vessels and spreads to aerial parts of the plant through the vascular system where it multiplies extensively and produces large amounts of exopolysaccharide (EPS) [9]. To cope with these various soil and plant microenvironments, *R*. *solanacearum* has evolved a complex regulatory network that senses key signals and triggers important physiological changes via global shifts in gene expression [10]. At the center of this virulence regulatory network is the global regulator PhcA, a LysR-type transcriptional regulator that controls expression of many genes [11].

Experimental evolution of the GMI1000 strain was conducted by serial passage experiments (SPE) from one plant individual to another in order to maintain the pathogen population in the same host for over 300 bacterial generations [6]. Five independent lineages of experimentally evolved clones were generated by conducting five parallel SPEs. Phenotypic analysis of the experimentally evolved clones demonstrated that almost 80% of them had an increased fitness in their experimental host compared to the ancestral GMI1000 clone. However, no increase in disease symptom rate was observed whether the experimental plant was a susceptible host or a tolerant host (on which *R*. *solanacearum* grows asymptomatically). Interestingly, genomic sequence analysis of the evolved clones revealed single nucleotide polymorphisms (SNPs) in the *efpR* gene in three of the five lineages evolved on bean, a tolerant host [6]. A reverse genetic approach in the GMI1000 strain confirmed that the SNPs detected in the *efpR* gene were associated with fitness gain on bean. This gene encodes a putative transcription regulator protein of 113 amino acids belonging to the HTH_XRE (Helix-Turn-Helix_Xenobiotic-Response-Element) superfamily. The *efpR* gene is highly conserved in all the 38 *R*. *solanacearum* strains representative of the species complex diversity sequenced to date, with more than 90% protein identity between all strains. This gene is also present in other β-proteobacteria such as *Burkholderia*, *Pandoraea* and *Bordetella* species with more than 80% protein identity. However, the function of the EfpR protein remains unknown.

The aim of the present study was to determine the functional impact of the mutations in the *efpR* gene, in order to understand how these mutations promote bacterial fitness *in planta*. We performed a transcriptomic profiling of the *efpR*-deleted mutant to assess the impact of EfpR on gene expression and the nature of its targets. We used a system biology approach using the recently published model of *R*. *solanacearum* [12] to predict the putative phenotypes associated with the large set of genes differentially expressed. Model predicts were used to drive functional analyses which then revealed that EfpR affects several metabolic pathways and important virulence traits such as motility and EPS production. We demonstrated that the SNPs selected in the *efpR* gene during the evolution experiment significantly alter the *efpR* expression. This work highlights the importance of EfpR as a global regulator which coordinates the expression of virulence functions to the metabolic status of the bacterial cell.

## Results

### Mutations in *efpR* provide a fitness advantage on different plant species

We previously identified three SNPs in the *efpR* gene from bacterial populations evolved on bean plants and these mutations were associated to the fitness gain on this host [6]. We investigated whether mutations in *efpR* also occurred in populations evolved in plant species other than bean. In a previous work, mutations in *efpR* were not detected in three independent populations propagated on tomato (populations A, B and E) and in three independent populations propagated on cabbage (populations A, B and C) [6]. Here, we conducted further analyses in 14 additional independent populations (populations C and D propagated on tomato, populations D and E propagated on cabbage, five independent populations propagated on eggplant and five independent populations propagated on melon). This analysis was conducted using a similar PCR / sequencing approach on 96 clones randomly isolated from each population as previously described [6]. Three new SNPs in *efpR* resulting in non-synonymous mutations were detected: a R97Q mutation in tomato_[population C]_, a R98W mutation in eggplant_[population A]_, and a F44C mutation in melon_[population B]_. This observation suggested that mutations in *efpR* were not solely associated to a fitness gain on bean, but also on additional hosts.

We tested this hypothesis by determining if the *efpR* mutation D49N selected on bean also conferred a fitness gain when bacteria were inoculated on other hosts (tomato and cabbage), using the same conditions as previously reported for bean [6]. Competition assays were performed using the Δ*efpR* mutant (GRS704), the allelic mutant *efpR* ^D49N^ (GRS886) and the complemented strain (GRS705) (Table 1). The competitive index (CI) was determined for each strain after two passages in plant stem (see Materials and Methods). The results showed that both the Δ*efpR* mutant and the allelic mutant *efpR* ^D49N^ had a mean CI significantly superior to the mean CI with the complemented strain in tomato (Wilcoxon test, *p*-value = 0.016 and 0.016, respectively) and in cabbage (Wilcoxon test, *p*-value = 0.028 and 0.016, respectively) (S1 Fig). These results demonstrated that both the deletion of *efpR* and the bean-*efpR* allele (*efpR* ^D49N^) also improve bacterial fitness on other plants in addition to bean.

**Table 1:** Bacterial strains used in this study.

Strain ID	Strain name	Relevant characteristic(s)	Reference or source
GMI1000	WT	Wild-type strain GMI1000	[13]
GRS704	Δ*efpR*	GMI1000, Δ*efpR*, Sp^R^	[6]
GRS705	Δ*efpR*::*efpR*	GMI1000, Δ*efpR*::*efpR* ^WT^, Sp^R^Gm^R^	[6]
AG87	c1_bean_	GMI1000 derived clone, after 26 SPE on bean, carrying *efpR* ^P93Q^ mutation	[6]
AG93	d2_bean_	GMI1000 derived clone, after 26 SPE on bean, carrying *efpR* ^L2L^ mutation	[6]
AG98	e2_bean_	GMI1000 derived clone, after 26 SPE on bean, carrying *efpR* ^D49N^ mutation	[6]
GRS884	*efpR* ^P93Q^	GMI1000, Δ*efpR*::*efpR* ^P93Q^, Gm^R^	This study
GRS885	*efpR* ^L2L^	GMI1000, Δ*efpR*::*efpR* ^L2L^, Gm^R^	This study
GRS886	*efpR* ^D49N^	GMI1000, Δ*efpR*::*efpR* ^D49N^, Gm^R^	This study
GRS825	Δeps	GMI1000, Δ*epsB-D*	[12]
GMI1605	*phcA*::Ω	GMI1000, *phcA*::Ω, Sp^R^	[14]

Note. Sp^R^, Gm^R^ indicate resistances to spectinomycin and gentamicin, respectively. SPE: serial passages experiments.

### EfpR is a global regulator affecting gene expression involved in metabolic processes

The global transcriptomes of the WT (wild-type strain GMI1000) and the Δ*efpR* mutant were determined using an RNA-sequencing approach of strains grown in minimal media supplemented with 20 mM glutamate. All samples rendered between 10 and 15 million of *Ralstonia*-mapped reads, with the exception of one replicate for the Δ*efpR* mutant (i.e. GRS704.R1), which rendered 37 million mapped-reads.

Comparative analysis of RNA-seq data was conducted by considering differentially expressed genes (DEGs) as those producing a Benjamini-Hochberg (BH) adjusted *p*-value <0.05 and presenting an absolute Fold Change (|FC|) between strains >2. Using these cutoff values, we found that the total number of DEGs in the Δ*efpR* mutant compared to the WT strain was 877 genes, including 522 up- and 355 down-regulated genes (S1 Table). Those genes represent 17% of all GMI1000 predicted genes [13]. One third of the DEGs encoded hypothetical proteins, including 148 up- and 160 down-regulated genes, and 1/15 of the DEGs encoded predicted transcription factors, including 34 up- and 27 down-regulated genes (S1 Table). However, these proportions were not significantly more than the random expectation based on the proportion of hypothetical proteins or transcription factors in the GMI000 genome. Among the predicted transcription factors that were differentially expressed in the Δ*efpR* mutant, the RSc3149 gene, which was one of the highest up-regulated genes (Fold Change = 8.12), is an EfpR homolog with 79% protein identity with the EfpR protein sequence.

**Fig 1:**
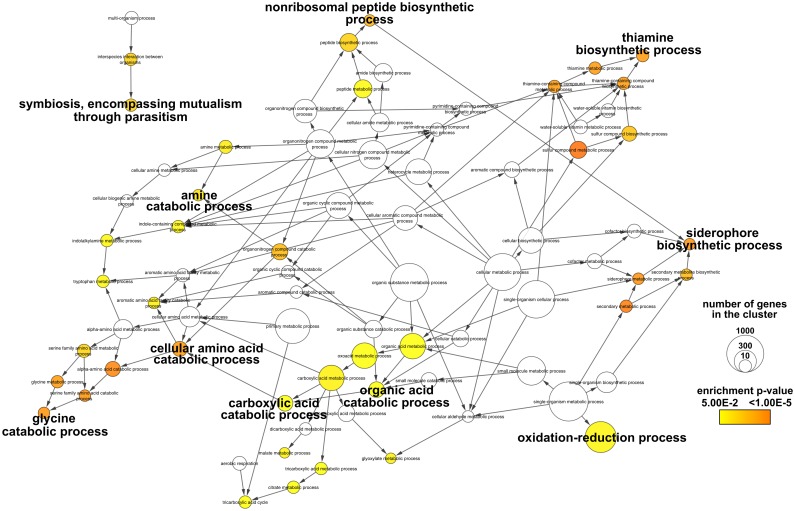
GO enrichment analysis of the up-regulated differentially expressed genes from Δ*efpR* mutant compared to the WT strain. Colors of the GO terms correspond to the significance of the *p*-value. The size of the node is proportional to the number of genes classified in the GO terms. We highlighted the main GO terms significantly enriched.

In order to get insights into the biological functions regulated by EfpR we performed a Gene Ontology (GO) enrichment analysis on DEGs (see Materials and Methods). Among the GO terms significantly enriched, metabolism-related terms were particularly abundant (Fig 1 and S2 Fig). Specifically, genes involved in several catabolic pathways, like amino acid and organic acid catabolism, were enriched in the EfpR-dependent up-regulated DEGs (Fig 1). In addition, genes involved in secondary metabolism (siderophore and thiamine cofactor biosynthesis) and in plant associated functions were also enriched in the EfpR-dependent up-regulated DEGs (Fig 1). In the EfpR-dependent down-regulated DEGs, genes involved in fatty acid biosynthesis and transporters were found to be enriched, as well as genes involved in cell cycle functions (S2 Fig).

### Analysis of EfpR-DEGs using genome-scale metabolic models predicted a role of EfpR in virulence and catabolism repression

The GO enrichment analysis indicated that metabolic pathways are particularly enriched in the EfpR regulon. Thus, we took advantage of the recently reconstructed genome-scale metabolic model of *R*. *solanacearum* [12] to infer the causal relationship between the EfpR-dependent DEGs (EfpR-DEGs) and metabolic processes.

**Fig 2:**
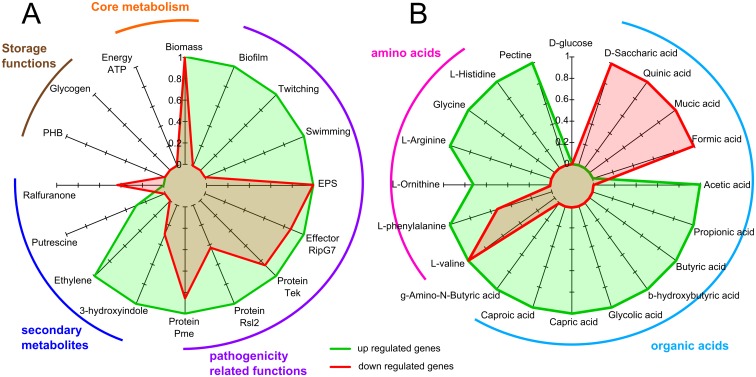
Predictions of phenotypes and catabolic pathways affected by EfpR-dependent DEGs (EfpR-DEGs) using flux balance analysis. Simulations were performed using the genome-scale metabolic network of *R*. *solanacearum*. (A) Probability of the phenotypes to be affected by EfpR-DEGs ranking from 0 (not affected in any environments tested) to 1 (affected in all environments tested). (B) Probability of the catabolic capacities to be affected by EfpR-DEGs. Green, up-regulated genes; red, down regulated genes. Pme: pectin methyl esterase secretion; Rsl2: mannose-binding lectin, cell attachment; EPS: exopolysaccharide production; PHB: polyhydroxybutyrate.

We first conducted phenotype simulations using flux balance analysis with the EfpR-DEGs as constraints (see Materials and Methods). A total of 16 phenotypes were tested in 7 different environments (see S2 Table for the list of phenotypes and environments tested). This analysis revealed that 13 out of the 16 phenotypes tested were affected by the EfpR-DEGs (Fig 2A). Among them are several virulence-related phenotypes like EPS production, swimming and twitching motility, and secretion of type III effectors, but also other biological functions like biomass production (Fig 2A). Only 3 phenotypes were not found to be affected by the EfpR-DEGs: the production of specific secondary metabolites, the biosynthesis of storage macromolecules and energy production (Fig 2A).

Since the GO analysis revealed enrichment in catabolic processes, we also tested the involvement of EfpR-DEGs in various catabolic capacities. We thus tested the catabolism of 58 substrates known to encompass around 90% of the substrates used by *R*. *solanacearum* [12]. This analysis revealed that the EfpR-DEGs were involved in the catabolism of 19 compounds out of the 58 tested (31%) (Fig 2B). These substrates were mainly amino acids and organic acids, in agreement with the GO enrichment analysis (Fig 2B). Among the 19 compounds for which catabolism was affected by the EfpR-DEGs, 15 were gained in the Δ*efpR* mutant due to up-regulated genes (Fig 2B). These predictions further suggest a role of EfpR in catabolic repression.

### Evidence that deletion of *efpR* enlarges the *R*. *solanacearum* catabolic capacities

In order to validate the phenotypes predicted to be EfpR-dependent, we conducted an analysis of the catabolic capacities of the Δ*efpR* mutant by using metabolic phenotype microarrays. This revealed that the Δ*efpR* mutant possessed a wider substrate usage capacity than the WT strain. Indeed the mutant was able to significantly better catabolize 65 substrates, but could also use 12 substrates not catabolized by the WT strain in these conditions (S3 and S4 Figs, S3 and S4 Tables). The metabolic capacities of the complemented strain were similar to those of the WT strain.

**Fig 3:**
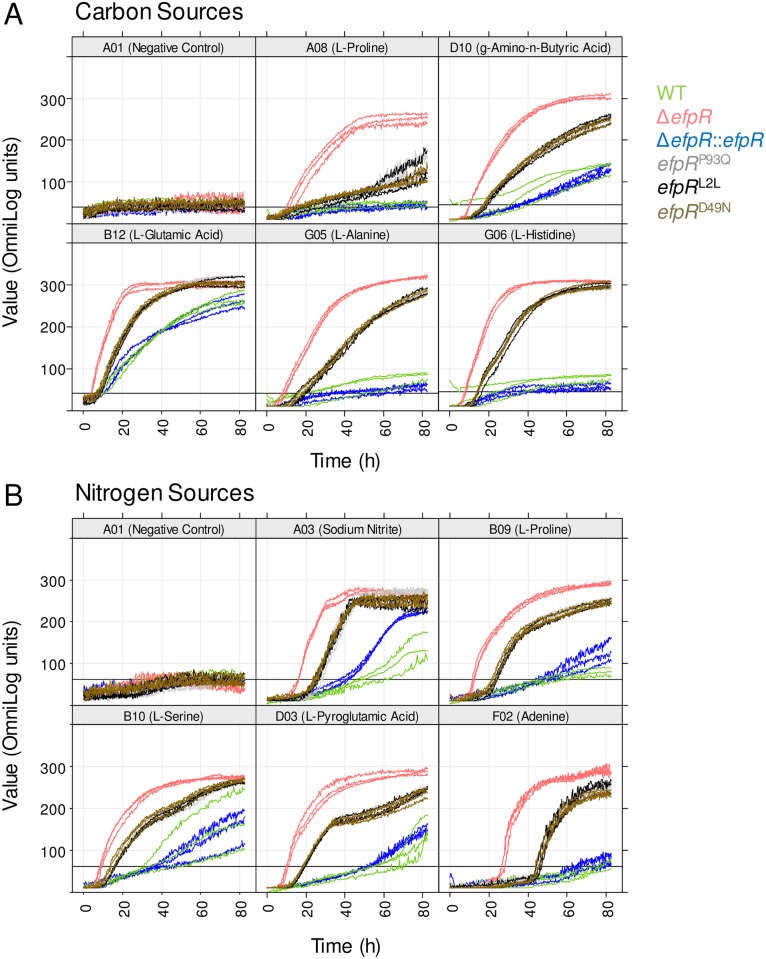
Phenotypic microarray curves for the Δ*efpR* mutant and the *efpR* allelic mutants compared to the WT strain. Phenotype Microarray data were collected over 82.5 hours at a temperature of 28°C on the Biolog plates PM01, PM02 and PM03. The respective curves from all six strains are superimposed. The x-axes show the measurement times in hours, the y-axes the curve heights in OmniLog units. In the caption of each panel the corresponding coordinate of the well is shown followed by the (A) carbon or (B) nitrogen source tested. Three replicates were performed. Data were treated with the R package opm.

**Fig 4:**
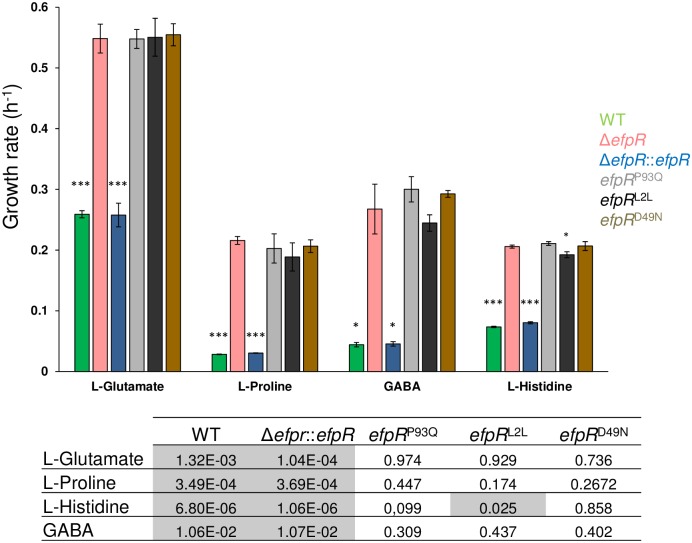
*In vitro* growth rate of *R*. *solanacearum* strains cultured in minimal medium supplemented with L-Glutamate, L-Proline, GABA or L-Histidine as sole carbon source. The bacterial growth (under shaking) has been followed during 50h by measurement of OD_600nm_ and then the growth rate determined during the exponential growth phase. Bars indicate standard deviation (Welch t-test, comparison with the Δ*efpR* strain, * *p*-value <0.05, ***p-value <0.001). The table gives the *p*-values from the Welch t-test comparing the growth rate of each tested strain with the growth rate of the Δ*efpR* strain.

Among the carbon and nitrogen sources that were poorly used by the WT strain, gamma-Amino-n-Butyric Acid (GABA) and L-Glutamate were significantly better used as carbon sources, likewise Sodium Nitrite, L-Serine and L-Pyroglutamic acid were significantly better used as nitrogen sources by the Δ*efpR* mutant (Fig 3). Even among the carbon and nitrogen sources that were not used by the WT strain, this analysis revealed that the Δ*efpR* mutant possessed a high efficiency to catabolize L-Alanine and L-Histidine as carbon sources, Adenine as nitrogen source and L-Proline both as carbon and nitrogen sources (Fig 3). The ability to catabolize L-Glutamate, L-Proline, L-Histidine and GABA was confirmed by investigating bacterial growth of the Δ*efpR* mutant and the complemented strain in comparison to the WT strain in minimal media supplemented with one of these four carbon sources. The results showed that the growth rate of the mutant on these four carbon sources was significantly increased compared to the WT (Welch t-test, *p*-value <1.32e-3, 3.49e-4, 6.80e-6, and 1.06e-2, respectively) and the complemented strains (Welch t-test, *p*-value <1.04e-4, 3.69e-4, 1.06e-6 and 1.07e-2, respectively) (Fig 4). All these data suggested that EfpR plays a major role in a catabolic repression operating in the bacterial cell.

### *efpR* loss of function enhances *R*. *solanacearum* swimming and twitching motility

The EfpR-DEGs analysis predicted that motility is affected in the mutant (Fig 2A). Motility is considered as one of the key factors determining the ability of the bacteria to colonize the roots of their host plants [15,16]. We compared the swimming and twitching motilities between the WT strain, the Δ*efpR* mutant and the complemented strain.

**Fig 5:**
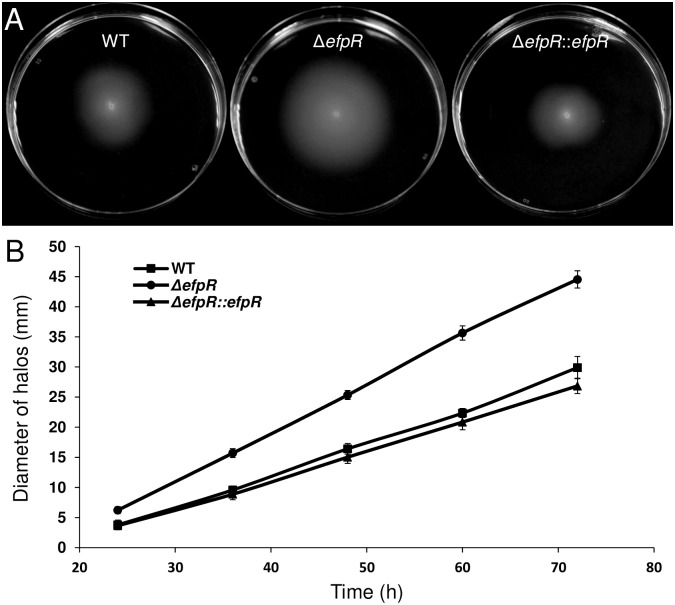
Deletion of *efpR* results in improved swimming motility of *R*. *solanacearum* GMI1000. (A) Swimming motility assay on MP minimal medium semi agar (0.3%) plates supplemented with L-Glutamate 20 mM. Representative pictures of swimming halos observed after incubation at 28°C during 72h. (B) The diameter of halos has been measured through the time. The experiment was repeated twice, with ten plates for each strain per replicate. Bars indicate standard deviation.

Comparison of the swimming halo diameters after 24, 36, 48, 60 and 72 hours between the different strains showed that the Δ*efpR* mutant had a significant wider mean diameter during the whole growth kinetic compared to the WT (Mann-Whitney test, *p*-value = 4.47e-8, 3.97e-8, 3.69e-8, 4.31e-8 and 4.84e-8, respectively) and compared to the complemented strain (Mann-Whitney test, *p*-value = 3.44e-8, 3.26e-8, 4.25e-8, 5.31e-8 and 5.32e-8, respectively) (Fig 5B). After 72 hours, the mean diameter of the Δ*efpR* mutant was 44.55 ± 1.43 mm while the mean diameters of the WT and the complemented strains were 29.9 ± 1.86 mm and 26.85 ± 1.28 mm, respectively (Fig 5A). From these data, we could estimate a speed of movement by swimming motility for each strain. The mean speed for the Δ*efpR* mutant was 0.81 ± 0.03 mm.h^-1^, significantly higher than the mean speed for the WT and the complemented strains (Welch t-test, *p*-value <2.2e-16), which were 0.54 ± 0.04 mm.h^-1^ and 0.49 ± 0.02 mm.h^-1^, respectively.

**Fig 6:**
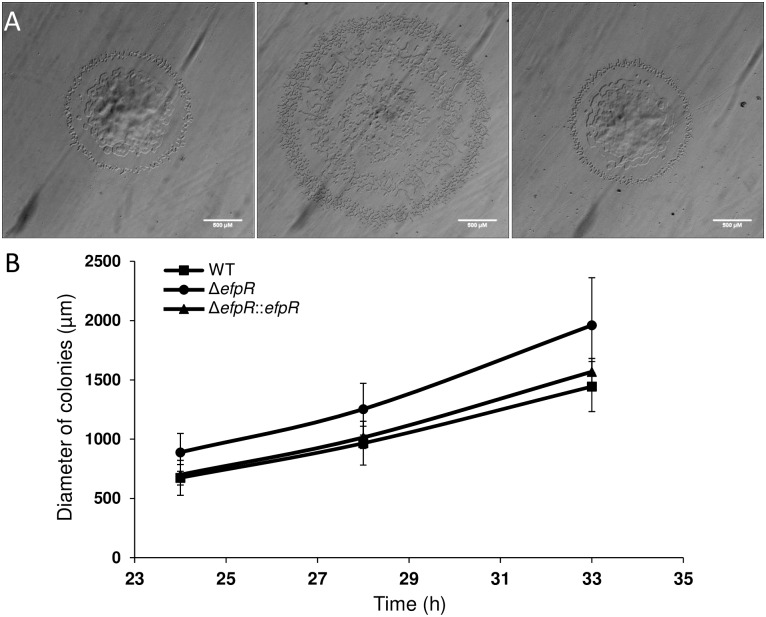
Deletion of *efpR* results in improved twitching motility of *R*. *solanacearum* GMI1000. (A) Typical morphology of *R*. *solanacearum* colonies after 33h incubation on BG medium plates (WT, Δ*efpR* and Δ*efpR*::*efpR* respectively from left to right). (B) The diameter of colonies has been measured through the time. The experiment was repeated twice, with twenty colonies for each strain per replicate. Bars indicate standard deviation.

Similar results were observed for twitching motility assays. The Δ*efpR* mutant had a significantly wider mean diameter than the WT strain (Welch t-test, *p*-value <1.93e-8, 1.36e-8 and 1.19e-9 after 24, 28 and 33 hours, respectively) (Fig 6). After 33 hours, the mean diameter of the Δ*efpR* mutant was 1961 ± 402 μm while the mean diameters of the WT and the complemented strains were 1443 ± 211 μm and 1568 ± 112 μm, respectively (Fig 6A). Again, from these data, we estimated the mean twitching motility speed for the Δ*efpR* mutant at 120 ± 29 μm.h^-1^, significantly higher than the mean speed for the WT and the complemented strains (Mann-Whitney test, *p*-value <1.16e-12 and 2.61e-5, respectively), which were 86 ± 9 μm.h^-1^ and 97 ± 6 μm.h^-1^, respectively.

### *efpR* loss of function alters EPS production in *R*. *solanacearum*

**Fig 7:**
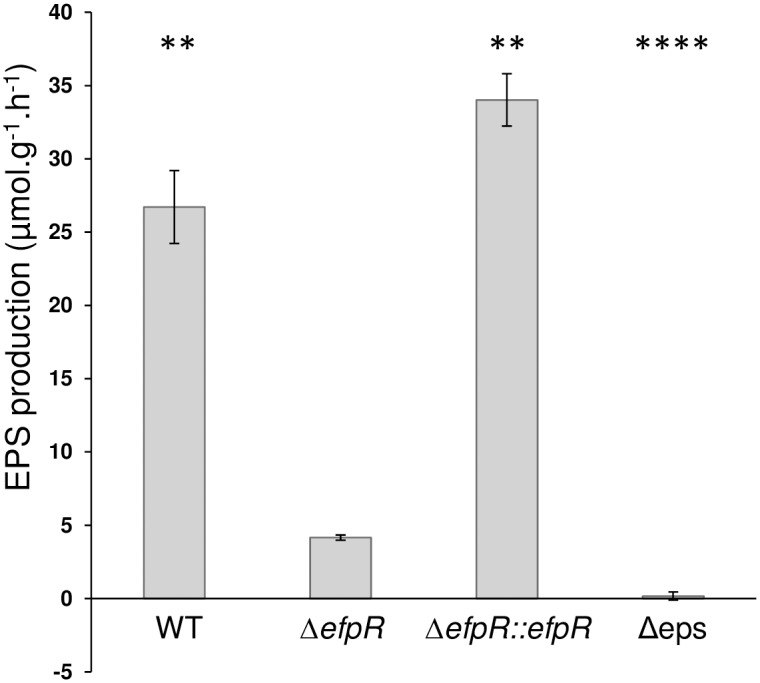
Deletion of *efpR* results in impaired EPS production of *R*. *solanacearum* GMI1000. Kinetic of EPS quantification has been done on minimal medium supplemented with 20mM glutamate, using the Elson Morgan Assay. Three biological repeats were conducted per strain. Bars indicate standard deviation (Welch t-test, comparison with the Δ*efpR* strain, ** *p*-value <0.01, ****p-value <0.0001).

The EfpR-DEGs analysis predicted that EPS production is affected in the Δ*efpR* mutant (Fig 2A). In addition, the colonies of the Δ*efpR* mutant were found to be less mucoid in appearance than those of the WT after 48 hours on agar plates, suggesting a decreased EPS production in the Δ*efpR* mutant. We quantified the flux of EPS production in cell culture supernatant in minimal medium with L-Glutamate as sole carbon source. The kinetics of hexosamine released in the Δ*efpR* mutant in comparison to the WT strain, the complemented strain and the Δeps mutant were quantified (see Materials and Methods). This analysis showed that the Δ*efpR* mutant produced 6.4 and 8.2 times less EPS than the WT and complemented strains, respectively (Welch t-test, *p*-value <3.84e-3 and 1.08e-3, respectively) (Fig 7). However, EPS biosynthesis was not completely abolished since the Δ*efpR* mutant still produced EPS compared to the Δeps mutant strain (Fig 7).

### The metabolic capacities of the *efpR* allelic mutants are similar to those of the *efpR* deleted mutant

In order to investigate the impact of the SNPs that appeared during experimental evolution on bean [6], we performed metabolic phenotype microarrays with the three *efpR* allelic mutants carrying the *efpR* base substitution as detected in the ‘bean’ evolved clones, namely the allelic mutants *efpR* ^P93Q^ (GRS884), *efpR* ^L2L^ (GRS885) and *efpR* ^D49N^ (GRS886) (Table 1).

Similarly to the Δ*efpR* mutant, microarray phenotyping revealed that the three allelic mutants possessed a wider substrate usage capacity than the WT strain, being able to significantly better catabolize 39, 42 and 38 substrates, with 4, 5 and 5 newly used substrates compared to the WT strain, respectively (S3 and S4 Figs, S3 and S4 Tables). The metabolic profiles of these three allelic mutants were similar to each other, but were intermediate to those of the Δ*efpR* mutant and the WT strain (Fig 3, S3 and S4 Figs).

The ability to catabolize L-Glutamate, L-Proline, L-Histidine and GABA was also confirmed by investigating bacterial growth of the three allelic mutants in comparison to the WT strain on minimal media supplemented with one of these four carbon sources (Fig 4). The results showed that the growth rates of the three allelic mutants on these four carbon sources were significantly increased compared to the WT and complemented strains (Welch t-test, *p*-value <0.05; Fig 4). However, these growth rates were not significantly different from the growth rate of the Δ*efpR* mutant (Welch t-test, *p*-value >0.05; Fig 4). Only the *efpR* ^L2L^ mutant had a growth rate on L-Histidine significantly lower than the Δ*efpR* mutant (Welch t-test, *p*-value = 0.025; Fig 4).

### Virulence of all *efpR* mutants is decreased on tomato plants

The virulence of the Δ*efpR* mutant, the complemented strain and the three *efpR* allelic mutants were tested on susceptible tomato plants in comparison to the virulence of the WT strain. Virulence assays were conducted on the susceptible tomato variety used during experimental evolution [6] and not on bean plants which remain asymptomatic after infection with strain GMI1000.

**Fig 8:**
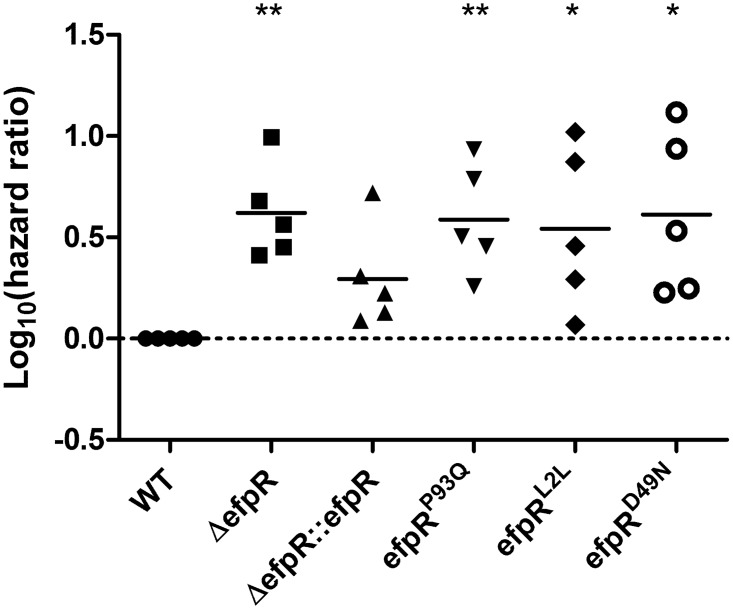
Virulence of *efpR* mutants and complemented strains vs the WT strain, on tomato plants. The data are represented as the hazard ratio between the tested strain and WT strain. The death rates of plants inoculated by soil drenching with the *efpR* mutants are significantly lower than the death rates of those inoculated with the WT strain, while the complemented mutant shows no significant difference with the WT strain (One sample t-test, comparison with the WT strain, * *p*-value <0.05, **p-value <0.01).

Fig 8 represents the log_10_(hazard ratio), the hazard ratio being the wilting rate for plants inoculated with the WT strain divided by the wilting rate of plants inoculated with mutant or complemented strains (see Materials and Methods for details). Five biological repeats were conducted, each repeat investigating the wilting of 16 tomato plants.

The obtained log_10_(hazard ratio) for the Δ*efpR* mutant and for the three allelic mutants *efpR* ^P93Q^, *efpR* ^L2L^, *efpR* ^D49N^ were significantly higher than zero (one sample t-test, *p*-value = 0.004, 0.0083, 0.0381 and 0.0287, respectively) (Fig 8). This result indicated that virulence of these four mutants was decreased on tomato plants compared to the WT strain, whereas the virulence of the complemented strain was not significantly different from the WT strain (one sample t-test, *p*-value = 0.06) (Fig 8).

### *efpR* expression is strongly altered in the *efpR* allelic mutants but not in a *phcA* mutant

**Fig 9:**
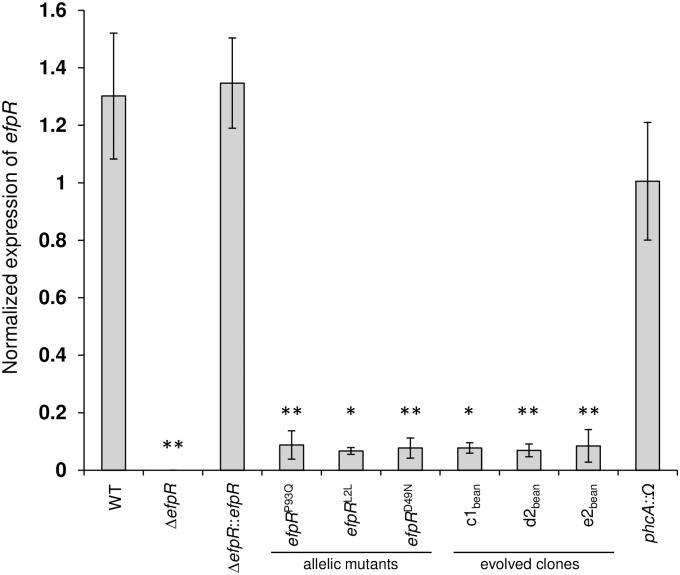
Different SNPs (synonymous and non-synonymous) in the *efpR* gene reduce the *efpR* gene expression during growth in minimal medium. Bacteria were grown in minimal medium supplemented with D-glucose (20 mM). Bacteria were harvested for total RNA extraction at OD_600nm_ ~ 0.5 and *efpR* mRNA were quantified using a qRT-PCR approach. Three biological repeats were conducted for each strain (two for the strain *efpR* ^P96Q^). Bars indicate standard deviation (Welch t-test, comparison with the WT strain, * *p*-value <0.05, **p-value <0.01).

Abundance of *efpR* mRNA in the three allelic *efpR* mutants was measured by qRT-PCR. Interestingly, this analysis revealed that the evolved allelic mutations, P93Q, L2L and D49N, induced a drastic reduction (>10 fold) in *efpR* mRNA level *in vitro* compared to the WT and complemented strains (Fig 9). This decrease of *efpR* expression was also true for three original ‘bean’ evolved clones carrying mutation in the *efpR* gene (Fig 9 and Table 1).

Finally, since PhcA also controls EPS production, twitching and swimming motilities [14–17], similarly to EfpR, we tested if the expression of *efpR* was dependent on PhcA. For that purpose, the *efpR* mRNA abundance was measured in a *phcA* mutant *in vitro* by qRT-PCR. This analysis revealed that the *efpR* mRNA abundance was similar in the *phcA* mutant and in the WT strain, thus demonstrating that the PhcA regulator didn’t control the expression of *efpR* in these conditions (Fig 9).

## Discussion

In this work, we investigated the role of the *efpR* gene, a gene involved in adaptation of *R*. *solanacearum* to various host plants, discovered during an experimental evolution where bacteria were maintained on plant lineages for more than 300 generations [6]. This former study showed that the fitness gain of the evolved bacteria was associated to SNPs within the *efpR* sequence. A similar fitness gain was obtained by deleting *efpR*. In the present study, we provide evidence that EfpR is mainly a repressor of gene expression (60% of the DEGs), suggesting that the mutations in *efpR* selected during the experimental evolution led to the de-repression of biological functions contributing to the observed fitness gain. Indeed, our data showed that EfpR regulates, directly or indirectly, a large number of genes (about 17% of the WT genes displayed a differential expression in the Δ*efpR* mutant). As a consequence, EfpR controls many metabolic pathways and virulence-associated traits. Similar changes in global gene expression through mutations affecting regulatory network components have been observed in evolution experiments conducted with bacteria and appear to be a first and rapid adaptive response to novel environmental conditions [18].

Based on the transcriptomic profiling of the Δ*efpR* mutant and flux balance analysis using the genome-scale model of *R*. *solanacearum*, we predicted several candidate functions to be controlled by EfpR. Phenotypic and functional characterization of the Δ*efpR* mutant first confirmed that this mutant has an expanded metabolic versatility since it is able to catabolize 65 carbon and nitrogen substrates better than the WT strain, including 12 substrates that the WT strain is unable to use in our conditions. Interestingly, this increased substrate usage pattern matches well with the list of carbon sources identified in the xylem of tomato plants [19]. Indeed, many amino acid identified in tomato xylem fluids, including L-Glutamate, L-Proline, L-Histidine and GABA, are better catabolized by the Δ*efpR* mutant. These results therefore establish that EfpR behaves as a global catabolic repressor in *R*. *solanacearum*, directly or indirectly down-regulating multiple metabolic pathways. However, we show that EfpR also controls functions associated to bacterial pathogenesis beyond central metabolism. An *efpR* mutant produces much less EPS than the WT strain, and EPS has been reported to be required for full virulence on plants [9]. This could explain the reduced pathogenicity of the Δ*efpR* mutant when inoculated on tomato plants. From the list of DEGs in the Δ*efpR* mutant, it is probable also that additional factors directly or indirectly linked to virulence are under the control of EfpR. Twitching and swimming motility were also shown to be repressed by EfpR, thus raising the hypothesis that the *efpR* mutant may have an increased invasive capacity during plant infection.

In order to get a better understanding of the impact of the *efpR* mutations that occurred during the evolution experiment, the three different SNPs from the evolved clones in bean were re-created in the WT strain. Interestingly, whatever the position of the SNP in the *efpR* gene, the three allelic mutants all shared very similar phenotypic characteristics that were very close, even if not identical, to the Δ*efpR* mutant. Similar enhanced growth rates were observed for the allelic and deleted mutants in presence of various carbon substrates and the allelic mutants were able to significantly catabolize a wider number of carbon and nitrogen substrates than the WT strain, even if this number was less than for the Δ*efpR* mutant. As observed with the Δ*efpR* mutant, virulence of the three allelic mutants was decreased on tomato plants. These results indicate that the *efpR* allelic mutants almost behave as loss-of-function mutants, which was further confirmed by showing that the three different SNPs all lead to a strong decrease in the expression of *efpR* (Fig 9). The residual *efpR* expression detected in the allelic mutants could explain the minor difference observed between the catabolic capacities of the allelic and deleted mutants.

Bacterial adaptation to novel habitats through loss-of-function mutations has recently been reported [20], with evidence that such mutations can favor adaptation to novel environments through regulatory and metabolic rewiring. Our findings support the view that the enlarged metabolic capacities of the *efpR* allelic mutants provide the fitness advantage occurring during growth in the plant xylem of different species and explains why such *efpR* mutations were rapidly fixed in the populations during the experimental evolution [6]. In addition, the strong impairment of *efpR* mutants to produce EPS could also contribute to this fitness gain since EPS biosynthesis represents a significant metabolic cost for the bacterial cell [12]. It cannot be excluded that the increased motility of *efpR* mutants also provides a competitive advantage over the WT strain by enabling a faster colonization of the host vascular system.

Modeling of the predicted EfpR structure reveals that the mutations detected in bean, tomato, eggplant and melon during the evolution experiment occurred in two main domains of the protein (S5 Fig): two SNPs (F44C and D49N) lead to changes in positions in the HTH domain just adjacent to the DNA binding site whereas three other SNPs are located in the exterior domain, inducing changes in charged amino acids (R97Q, R98W) or on the probable folding of the domain (P93Q). This cluster of mutations in this protruding domain is suggestive of a role in interacting with another protein or co-factor and/or that it is critical for EfpR stability. A third SNP is a synonymous mutation occurring in the second codon, presumably altering the gene functionality [21].

The experimental evolution scheme developed by Guidot and collaborators [6] was based on serial passages of bacterial populations which were collected from the xylem of infected stems at the onset of wilting symptoms and then stem-inoculated on the next plant. This experimental procedure therefore avoided the root infection step, which is the natural mode of entry of the soilborne *R*. *solanacearum* in its hosts. Our results showed that the *efpR* mutants are significantly reduced in virulence when using ‘natural’ soil drenching infection assays (Fig 8). This probably explains why *efpR* mutations which occurred several times during the experimental evolution might be counter-selected in nature. It is tempting to speculate that EfpR plays an important role during steps of the *R*. *solanacearum* life cycle (such as in the soil or during root infection) which were not reproduced during the evolution experiment and which may be advantageous for the pathogen in its natural context.

Altogether the results presented in this study establish that EfpR is a novel player of the complex regulatory network controlling *R*. *solanacearum* virulence in response to multiple environmental signals [11]. EfpR is presumably a key component of this network which tightly links the bacterial metabolism to virulence. Beyond EPS production and motility, the prediction of phenotypes affected by EfpR-DEGs suggests that additional functions such as biofilm formation, secondary metabolism and other virulence-associated proteins are also controlled by EfpR. The pleïotropic phenotype of the *efpR* mutant is reminiscent of the phenotype of another key *R*. *solanacearum* virulence regulatory gene, *phcA*, identified more than 25 years ago [22]. As *efpR*, a *phcA* mutant is defective for EPS production and many other virulence factors, and displays an hypermotile phenotype [22]. More recently, a *phcA* mutant was also found to have an expanded metabolic versatility [12], being able to metabolize an even wider repertoire of metabolic substrates than the *efpR* mutant. This phenotypic relatedness incited us to explore the existence of functional links between PhcA anf EfpR. Our results indicated that the expression of *efpR* is not significantly altered in a *phcA* mutant (Fig 9) and that *phcA* does not appear to be differentially regulated in the *efpR* mutant background. This suggests that if there is interplay between these two central regulators, this should operate at a post-transcriptional level. Future works will aim at understanding how PhcA anf EfpR jointly orchestrate a tight coupling between catabolic pathways (supporting *in planta* growth) and virulence functions, in a mechanism which appears to be driven by a resource allocation trade-off. The discovery of the role played by EfpR in this process also highlights how the experimental evolution approach helps to uncover a key regulatory component of *R*. *solanacearum* pathogenicity and adaptation to hosts.

## Materials and Methods

### Bacterial strains, plant material and growth conditions

*R*. *solanacearum* strains used in this study are described in Table 1. Strains were grown in complete BG medium or in MP minimal medium at 28°C [23]. The pH of the MP medium was adjusted to 6.5 with KOH. For agar plates, BG medium was supplemented with D-Glucose (5 g liter^-^1) and triphenyltetrazolium chloride (0.05 g liter^-^1). The MP medium was supplemented with various carbon sources (L-Glutamate, D-Glucose, L-Proline, gamma-Amino-n-Butyric Acid (GABA) or L-Histidine) at 20 mM final concentration. When needed, antibiotics were added to the media at the following final concentrations (mg liter^-^1): spectinomycin, 40; gentamicin, 10.

The plants used in this study were tomato (*Solanum lycopersicum* var. Super Marmande), eggplant (*S*. *melongena* var Zebrina), cabbage (*Brassica oleracea* var. Bartolo) and melon (*Cucumis melo* var. Vedrantais). Four- to five-week-old plants were used for the inoculations. Plant experiments were conducted in a growth chamber under the following conditions: 75% humidity, 12h light 28°C, 12h darkness 27°C.

### Detection of mutations in *efpR*

Mutations in *efpR* in populations evolved in plant species different from bean was determined by PCR amplification and Sanger sequencing using the primers RSc1097-L and RSc1097-R (S5 Table) from cell suspension of 96 clones randomly isolated in each population as described in Guidot et al. [6].

### Bacterial competition assays *in planta*

To monitor the *in planta* fitness of the *efpR* mutants, we conducted competition assays as previously described [6]. Briefly, a mixed inoculum at a 10^6^ CFU/ml concentration, was prepared, containing equal CFU of the mutant (spectinomycin or gentamicin resistant) and the WT strain, and 10 μl was used to inoculate the stem of plants. After 5 days for the tomato susceptible plants and after 15 days for the cabbage tolerant plants, the bacteria were recovered from the stem, serial diluted and plated onto BG medium with and without spectinomycin or gentamicin. The competitive index (CI), which is the mutant/WT ratio recovered from the plant stem divided by the mutant/WT ratio in the inoculum, was determined after two passages in plant stem (S1 Fig). A total of five replicates were performed for each strain.

### Construction of base-substitution mutants in the *efpR* gene

Base substitution mutants at the *efpR* locus were created using the natural co-transformation ability of *R*. *solanacearum* and following an adapted protocol of the multiplex genome editing by natural transformation (MuGENT) described in Dalia et al. [24]. Briefly, PCR primers were designed to amplify 3kb arms of homology from either side of the SNPs (S5 Table). Then, high fidelity PCR has been performed on the genomic DNA of clones evolved in bean. The PCR products were then concentrated by sodium acetate precipitation and served as unselected products. The selected product was carried by an insertional plasmid pAGSI (a pRCG-GWY-based plasmid [25]) linearized by *Sca*I. Natural transformation of *R*. *solanacearum* was performed, as previously described [26], using 300 ng of the selected marker and 1 μg of unselected marker added to 50 μl of competent cells. *R*. *solanacearum* transformants were selected after growth on BG medium supplemented with gentamicin and then the co-transformants were validated by PCR amplification and Sanger sequencing using the primers RSc1097-L and RSc1097-R (S5 Table). The resulting *efpR* allelic mutants were called *efpR* ^P93Q^, *efpR* ^L2L^ and *efpR* ^D49N^ mutants in the present paper (Table 1).

### RNA extraction, depletion of rRNA and sequencing

Total RNA was extracted from the WT GMI1000 strain and the Δ*efpR* mutant strain growing in MP medium supplemented with L-Glutamate 20 mM at comparable cell densities (OD_600nm_ ~ 0.5). Three biologically independent experiments were conducted for the GMI1000 strain and two for the Δ*efpR* mutant. Before RNA extraction, the bacterial culture was first stopped by mixing 1 ml ethanol/phenol (95:5) to 20 ml of culture during 3 min using a vortex. The culture was then centrifuged at 4000 x g for 10 min at 4°C and the pellet was resuspended in 200 μl H_2_O RNase free for total RNA extraction. Total RNA was isolated and depleted of ribosomal RNAs as previously described [27]. The oligonucleotide sets used for the ribosomal RNA depletion were specifically designed to target *R*. *solanacearum* rRNAs (S5 Table).

Oriented paired-end RNA sequencing (2x100 bp) was carried out by Fasteris (Fasteris SA, Plan-les-Ouates, Switzerland), using an Illumina Hiseq 2000 and the procedures recommended by Illumina, with adaptors and amplification primers designed by Fasteris. The size of selected inserts was 150–250 bp. Libraries were sequenced in paired-end. Two technical repeats per RNA sample were performed.

### Mapping and analysis of RNAseq data

Read pairs were mapped using the glint software (http://lipm-bioinfo.toulouse.inra.fr/download/glint/) with parameters set as follows: matches ≥40 nucleotides, with ≤3 mismatches, only best-scoring hits taken into account. Ambiguous matches (same best score for several read-pairs) were removed. Finally, between 8.2 and 23.4M non ambiguous read-pairs were obtained. Mapped reads were imported into R environment. The package HTSFilter was used to eliminate very low-expressed genes from the analysis. A total of 5157 out the predicted 5307 genes were thus kept in. R package DESeq2 was used to normalize and complete the differential analysis by conducting the built-in Wald test [28]. The *p*-values thus obtained were adjusted for multiple comparisons using the Benjamini-Hochberg (BH) method [29]. Genes with a BH-adjusted *p*-value <0.05 and Fold Change |FC|>2 between strains were taken into further consideration in this work.

### Gene ontology analysis

We collected Gene Ontology from the Gene Ontology (GO) Consortium website (http://geneontology.org/) and assigned the GO annotation to *R*. *solanacearum* proteome using InterProScan [30]. Enrichments analysis of GO terms filed under Biological Process were separately conducted for up- and down-regulated genes using the R package topGO. Terms producing a *p*-value <0.05 after the Fisher exact test were taken into further consideration. GO enrichment visualization was performed using Cytoscape 3.2.1 and plug-in the Biological Networks Gene Ontology [31].

### Prediction of EfpR phenotypes from DEGs

Predictions of bacterial phenotypes were performed by Flux Balance Analysis [32] using the genome-scale metabolic network model of *R*. *solanacearum* GMI1000 [12]. Simulations were conducted using the software FlexFlux [33]. Predictions of the phenotypes feasibility were done by optimizing the metabolic fluxes corresponding to each phenotype using the DEGs as constraint. Two sets of DEGs constraints were used corresponding to the up-regulated genes set and the down-regulated genes set. Indeed, the states of the genes in the DEGs sets were set to 0. This simulated the behavior of genes which are not expressed or deleted. Thus, by doing so, we assessed the essentiality of the DEGs sets for the various phenotypes. Predictions were conducted considering various environmental conditions as constraints. These environmental conditions correspond to different composition of the surrounding of the cells, *in vitro* or *in planta*. Hence, uptake of a substrate was possible, i.e. exchange fluxes can be >0, only if the compounds are present in the environment. A set of 7 environmental conditions were tested (S2 Table). Then, the probability of the phenotypes to be affected by the EfpR-DEGs sets was assessed by calculating the number of environments in which the phenotype was affected on the number of environments tested.

### Prediction of catabolic repression by EfpR

Prediction of bacterial catabolism was performed by Flux Balance Analysis as described previously. The objective function optimized was the ATP hydrolysis flux (Non-growth associated maintenance). The environmental conditions tested correspond to the Biolog phenotype microarray environments PM01 and PM02. In those conditions only one carbon substrate is available allowing simulating a total of 190 catabolic capabilities corresponding to more than 90% of the *R*. *solanacearum* versatility [12].

### Phenotype microarray

Phenotypic microarrays were performed using Biolog Phenotype Microarray plates PM01, PM02 and PM03 according to the constructor protocol with the following modifications. Before inoculation of Biolog fluid IF-0, the cells were collected from a static culture on plate containing agar BG medium and resuspended in 15 ml sterile H_2_O supplemented with D-glucose at 20 mM with an OD_600nm_ between 0.3 and 0.5. The bacterial cells were then incubated during 6h at 28°C and 180 rpm agitation. This step, which starved the bacteria for nitrogen, phosphorus and sulfate, was found to reduce the background observed in PM03. D-glucose at 20 mM concentration was used as carbon source for the inoculation of the PM03 plate. The measurements were recorded on Omnilog reader (Biolog) during 82.5 hours. Three biological replicates were performed. Data were analyzed and statistic calculated using the R software package opm [34].

### Monitoring *in vitro* growth rates

Overnight cultures grown at 28°C and 180 rpm shaking in minimal medium supplemented with L-Glutamate 20 mM were used to inoculate three replicates of 200 μl of fresh minimal medium supplemented with L-Glutamate, L-Proline, GABA or L-Histidine 20 mM with an initial OD_600nm_ at 0.05. Bacterial growth was performed in 96-well microplates and monitored using a microplate spectrophotometer (FLUOstar Omega, BMG Labtech, Offenburg, Germany). The incubation temperature was fixed at 28°C and the microplates were shaken continuously at 700 rpm using the linear-shaking mode. Measures of OD_600nm_ was performed every 5 min during 50 hours. Three biological repeats were performed. Differences between *in vitro* growth rates were tested using a Welch t-test performed in the R statistical software.

### Twitching motility test

Bacteria were grown in BG medium for 1 day at 28°C. The cultures were then diluted and plated on BG agar medium supplemented with 20 mM Glucose to have isolated colonies. The colony diameter was compared after 24, 28 and 33 hours of incubation at 28°C. Colonies were examined for twitching motility under a stereomicroscope (Leica MZ FLIII). Colonies with layered edges and multiple irregular projections (‘spearheads’) are typical of migration of cells over the agar surface by twitching motility (Fig 6A) [15]. Digital images were acquired using a colour CCD camera (Leica DFC420) and saved as uncompressed TIFF files. The experiment was repeated twice, with twenty colonies for each strain per replicate. Differences between colonies diameters were tested using a Welch t-test performed in the R statistical software.

### Swimming motility test

Bacteria were grown in MP minimal medium supplemented with L-Glutamate 20mM for 1 day at 28°C. The bacterial culture (diluted to 1.10^8^ CFU/ml) was stab-inoculated into the agar of freshly poured MP minimal medium semi agar (0.3%) plates supplemented with L-Glutamate 20 mM. The diameter of the swimming halo was then measured after 36, 48, 60 and 72 hours of incubation at 28°C. The experiment was repeated twice, with ten plates for each strain per replicate. Differences between swimming halo diameters were tested using a Mann-Withney test performed in the R statistical software.

### EPS quantification

Bacteria were grown in MP minimal medium supplemented with L-Glutamate 20mM for 1 day at 28°C then centrifugated (5000 x g) during 15 min to remove EPS. Pellets were resupended in fresh MP minimal medium supplemented with L-Glutamate 20mM and then diluted to have an OD_600nm_ = 0.2. After 0, 4.33, 6.5, 8.83 and 11h of incubation at 28°C, 2ml of culture has been filtrated (0.22 μM) and EPS production of *R*. *solanacearum* cells was assayed by the Elson Morgan method as described in Peyraud et al. [12].

### Virulence tests

Bacteria were grown overnight in BG liquid medium with appropriate antibiotics. Virulence tests were conducted on 16 4-week-old tomato plants grown in 30x30 cm trays filled with compost. The 16 tomato plants were inoculated by soil drenching with 500 ml of a 5.10^7^ CFU/ml bacterial suspension. Plant symptoms were scored daily using a disease-index scale ranging from 0 (no symptoms) to 4 (complete wilting) as described in Poueymiro et al. [35]. This virulence test was repeated five times for each strain.

In order to compare the virulence of the *efpR* mutants, the complemented strain and the WT strain on tomato plants, the disease scoring was transformed into binary data, with a disease index below 3 corresponding to 0 and a disease index equal to or higher than 3 corresponding to 1. This transformation was performed in order to construct survival curves and to apply the survival analysis statistical protocols [36]. The hazard is defined as the slope of the survival curve and is a measure of how rapidly plants are dying. The hazard ratios comparing the survival curve of each *efpR* mutant or complemented strain to the survival curve of the WT strain were calculated by Graphpad Prism 5.0 software and the log_10_(hazard ratio) transformation was used to analyze the data as previously described [37].

### qRT-PCR

Total RNA were isolated using TRIzol Reagent (life technologies) followed by RNeasy MiniElute Cleanup Kit (Qiagen). To avoid contamination by genomic DNA each sample was treated with the TURBO DNA-free Kit (life technologies). The reverse transcription was performed on 1 μg of total RNA using the Transcriptor Reverse Transcriptase (Roche) with random hexanucleotides primers. Quantitative PCRs were performed on a Roche LightCycler480 using The LightCycler 480 SYBR Green I Master (Roche). Cycling conditions were as follows: 95°C for 5 min, 45 cycles at 95°C for 15 s, 60°C for 20 s and 72°C for 20 s. The specificity of each amplicon was validated with a fusion cycle. The mean efficiency of each amplicon group was determined using the LinRegPCR software [38]. The expression of *efpR* was normalized using the geometric average of three selected reference genes (RSc0403, RSc0368 and RSp0272) for each sample [39]. All kit and reagents were used following the manufacturer’s recommendations. The primer sets used in the experiments are listed in S5 Table.

## Supporting Information

S1 Fig Competitive index (CI) values of the Δ*efpR* mutant, the complemented strain and the ‘bean’ allelic mutant *efpR* ^D49N^ after two passages into tomato or cabbage plants. (A) Serial Passage Experiment (SPE) scheme starting with a mixed inoculum of the *efpR* mutant and the WT strain in the same proportion. (B) CI values obtained after two passages into tomato or cabbage plants. The CI values were compared with the CI obtained with the Δ*efpR*::*efpR* control strain using a Wilcoxon test (* < 0.05).
(PPTX)

S2 Fig GO enrichment analysis of the down-regulated differentially expressed genes from Δ*efpR* mutant compared to the WT strain (see Fig 1 for legend). (TIF)

S3 Fig Phenotype Microarray scatter plot of the sources usage by the WT strain and the *efpR* mutants. Phenotype microarray data were collected upon 82.5 hours at a temperature of 28°C on the Biolog plates PM01 (A), PM02 (B) and PM03 (C). At least three replicates were performed. Data were treated with the R package opm.
(PDF)

S4 Fig Phenotype Microarray heat map of the area under the curve value for the WT strain and the *efpR* mutants. Phenotype microarray data were collected upon 82.5 hours at a temperature of 28°C on the Biolog plates PM01 (A), PM02 (B) and PM03 (C). At least three replicates were performed. Data were treated with the R package opm.
(PDF)

S5 Fig Position of the SNPs observed in the evolved clones on the predicted EfpR structure. The structure of EfpR bounded to DNA was predicted using I-TASSER [40] and the drawing was done using UCSF Chimera. Accuracy of the I-TASSER prediction corresponds to a C-score of -1.18, a TM-score of 0.57± 0.15, and a RMSD of 6.6±4 Å. The EfpR surface is drawn in white, the binding site in the Helix-Turn-Helix (HTH) domain is in dark blue, and the DNA is in light blue. The binding site corresponds to the NCBI Blast prediction: the sequence specific DNA binding site contains the residues 39, 40, 51, 54, 58, 59; the non-specific DNA binding site residues are 29, 33, 58 and the salt bridges involved residues 57, 32. The mutated (non-synonymous) residues are colored in green (P93Q and D49N) for mutations isolated from bean, in red (R97Q) for Tomato, purple (R98W) for Eggplant and orange (F44C) for Melon.
(TIFF)

S1 Table RNA-seq data for all WT GMI1000 strain and Δ*efpR* mutant transcripts detected in minimal media supplemented with glutamate. (XLSX)

S2 Table List of conditions and list of phenotypes used in this study for the simulations and simulation results. (XLSX)

S3 Table Phenotype Microarray area under the curve (AUC) raw data for the Δ*efpR* mutant and the three *efpR* allelic mutants compared to the WT strain. The data were collected upon 82.5 hours at a temperature of 28°C on the Biolog plates PM01, PM02 and PM03. The AUC values were extracted with the R package opm. Three biological repeats are represented for each strain.
(XLSX)

S4 Table Phenotype Microarray area under the curve (AUC) data analysis for the Δ*efpR* mutant and the three *efpR* allelic mutants compared to the WT strain. The mean AUC corrected and the standard deviation from 3 biological repeats were determined for each strain and each source tested on the plates PM01, PM02 and PM03. The substrate usage is represented and the differential usage was tested using the Student t-test.
(XLSX)

S5 Table Primers used in this study. (PPTX)
