# Supplementary material for: Enhanced in planta Fitness through Adaptive Mutations in EfpR, a Dual Regulator of Virulence and Metabolic Functions in the Plant Pathogen Ralstonia solanacearum
Source: PLoS Pathog. 2016 Dec 2;12(12):e1006044. doi: 10.1371/journal.ppat.1006044 (PMC5135139; doi:10.1371/journal.ppat.1006044)
Supplement: S1 Fig — (A) Serial Passage Experiment (SPE) scheme starting with a mixed inoculum of the efpR mutant and the WT strain in the same proportion. (B) CI values obtained after two passages into tomato or cabbage plants. The CI values were compared with the CI obtained with the ΔefpR::efpR control strain using a Wilcoxon test (* < 0.05). (PPTX) [file ppat.1006044.s001.pptx]

## Slide 1
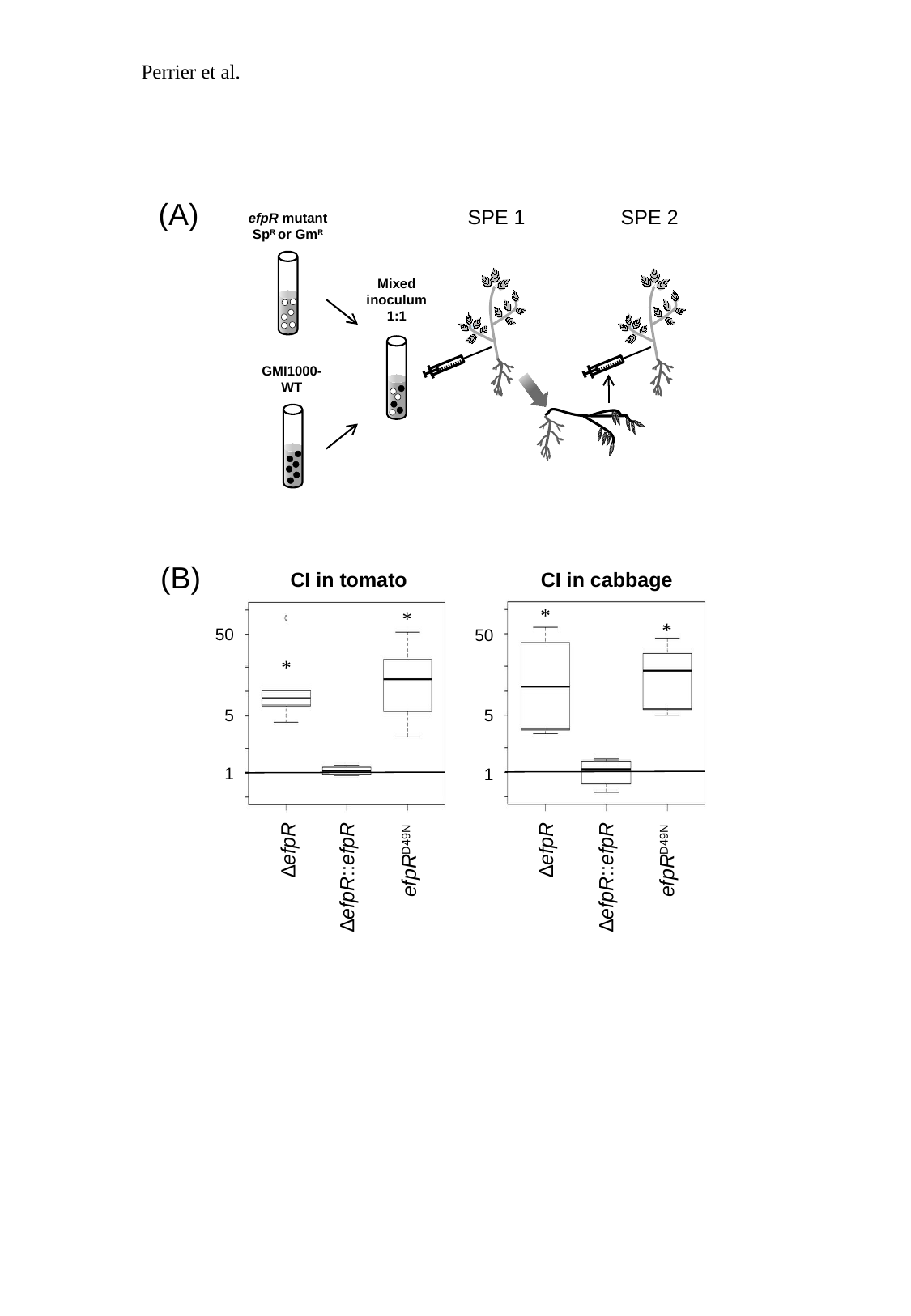

Perrier et al.
(A)
SPE 1
SPE 2
efpR mutant SpR or GmR
Mixed inoculum
1:1
GMI1000-WT
(B)
CI in tomato
CI in cabbage
*
*
*
50
50
*
5
5
1
1
∆efpR
∆efpR
efpRD49N
efpRD49N
∆efpR::efpR
∆efpR::efpR
