# Supplementary figures and images for: Enhanced in planta Fitness through Adaptive Mutations in EfpR, a Dual Regulator of Virulence and Metabolic Functions in the Plant Pathogen Ralstonia solanacearum
Source: PLoS Pathog. 2016 Dec 2;12(12):e1006044. doi: 10.1371/journal.ppat.1006044 (PMC5135139; doi:10.1371/journal.ppat.1006044)

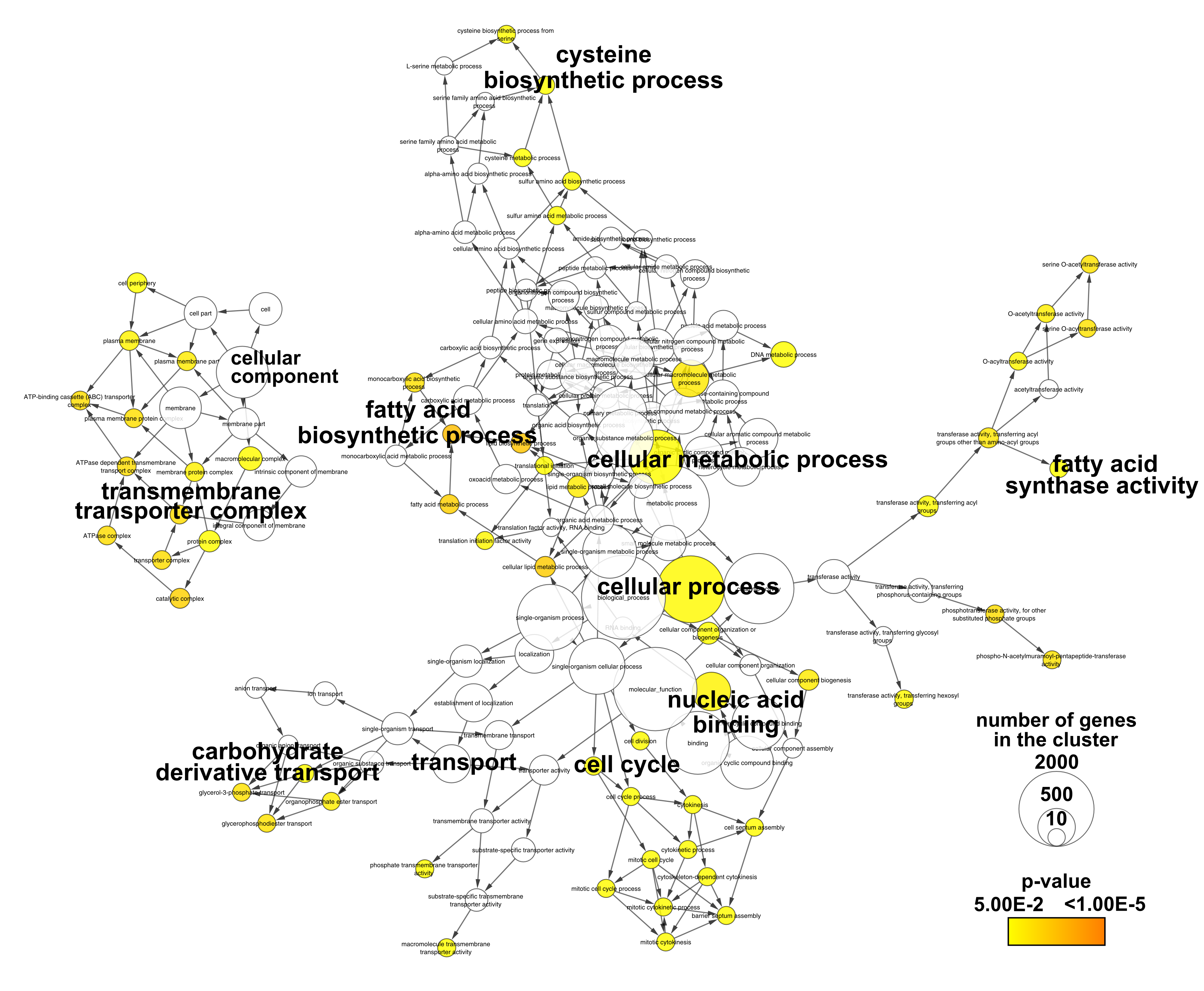

Supplement: S2 Fig — (TIF) [file ppat.1006044.s002.tif]

A

PM01 - Carbon sources usage

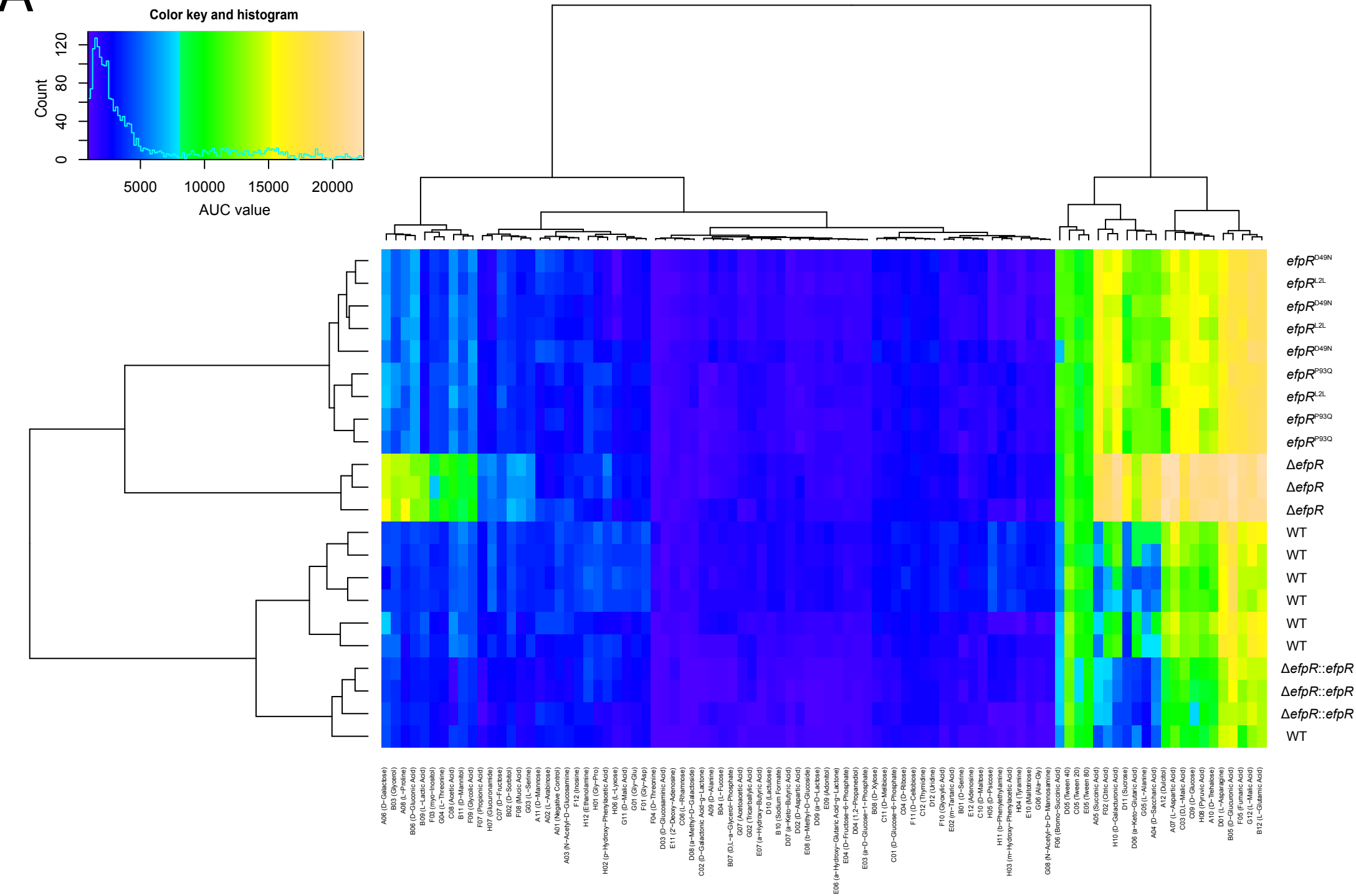

## B PM02 - Carbon sources usage

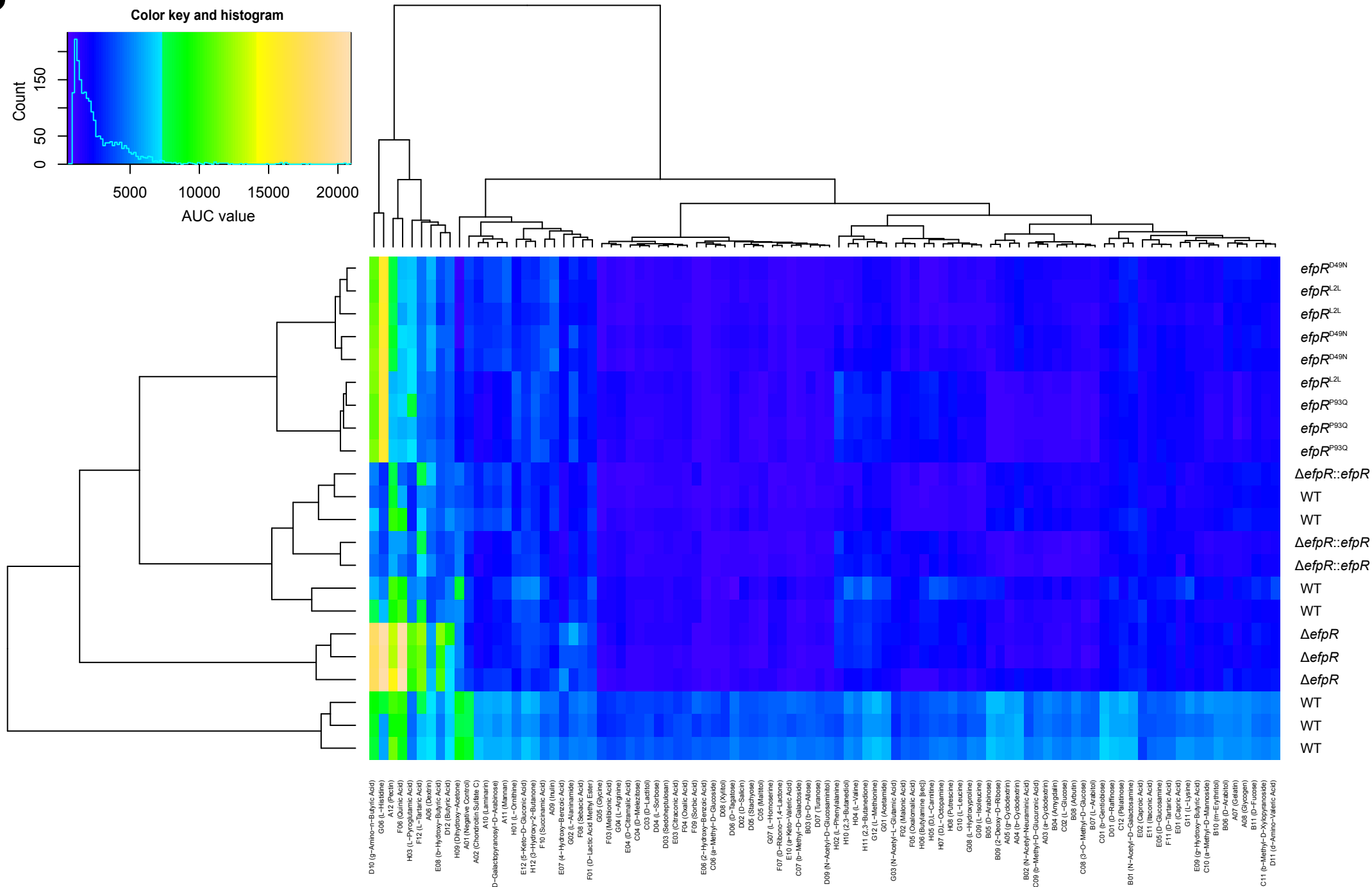

## C

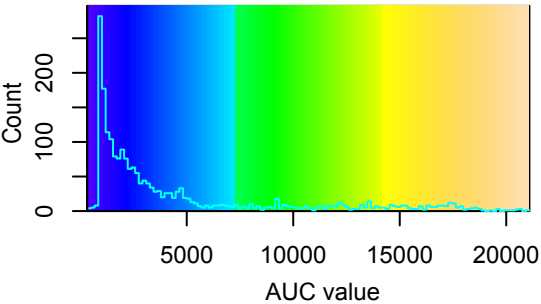

Supplement: S4 Fig — Phenotype microarray data were collected upon 82.5 hours at a temperature of 28°C on the Biolog plates PM01 (A), PM02 (B) and PM03 (C). At least three replicates were performed. Data were treated with the R package opm. (PDF) [file ppat.1006044.s004.pdf]

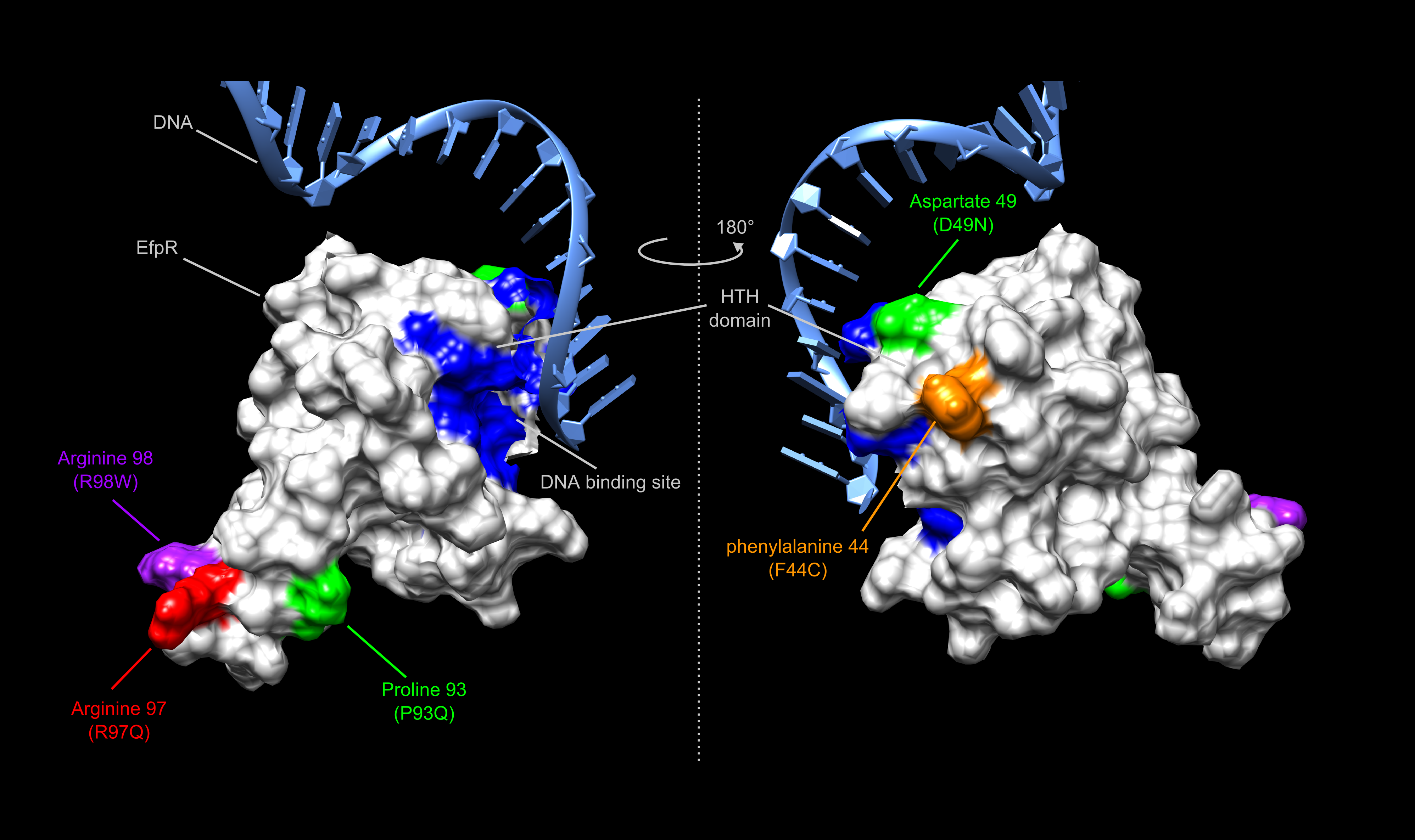

Supplement: S5 Fig — The structure of EfpR bounded to DNA was predicted using I-TASSER [40] and the drawing was done using UCSF Chimera. Accuracy of the I-TASSER prediction corresponds to a C-score of -1.18, a TM-score of 0.57± 0.15, and a RMSD of 6.6±4 Å. The EfpR surface is drawn in white, the binding site in the Helix-Turn-Helix (HTH) domain is in dark blue, and the DNA is in light blue. The binding site corresponds to the NCBI Blast prediction: the sequence specific DNA binding site contains the residues 39, 40, 51, 54, 58, 59; the non-specific DNA binding site residues are 29, 33, 58 and the salt bridges involved residues 57, 32. The mutated (non-synonymous) residues are colored in green (P93Q and D49N) for mutations isolated from bean, in red (R97Q) for Tomato, purple (R98W) for Eggplant and orange (F44C) for Melon. (TIFF) [file ppat.1006044.s005.tiff]
