# Supplementary material for: Enhanced in planta Fitness through Adaptive Mutations in EfpR, a Dual Regulator of Virulence and Metabolic Functions in the Plant Pathogen Ralstonia solanacearum
Source: PLoS Pathog. 2016 Dec 2;12(12):e1006044. doi: 10.1371/journal.ppat.1006044 (PMC5135139; doi:10.1371/journal.ppat.1006044)
Supplement: S3 Fig — Phenotype microarray data were collected upon 82.5 hours at a temperature of 28°C on the Biolog plates PM01 (A), PM02 (B) and PM03 (C). At least three replicates were performed. Data were treated with the R package opm. (PDF) [file ppat.1006044.s003.pdf]

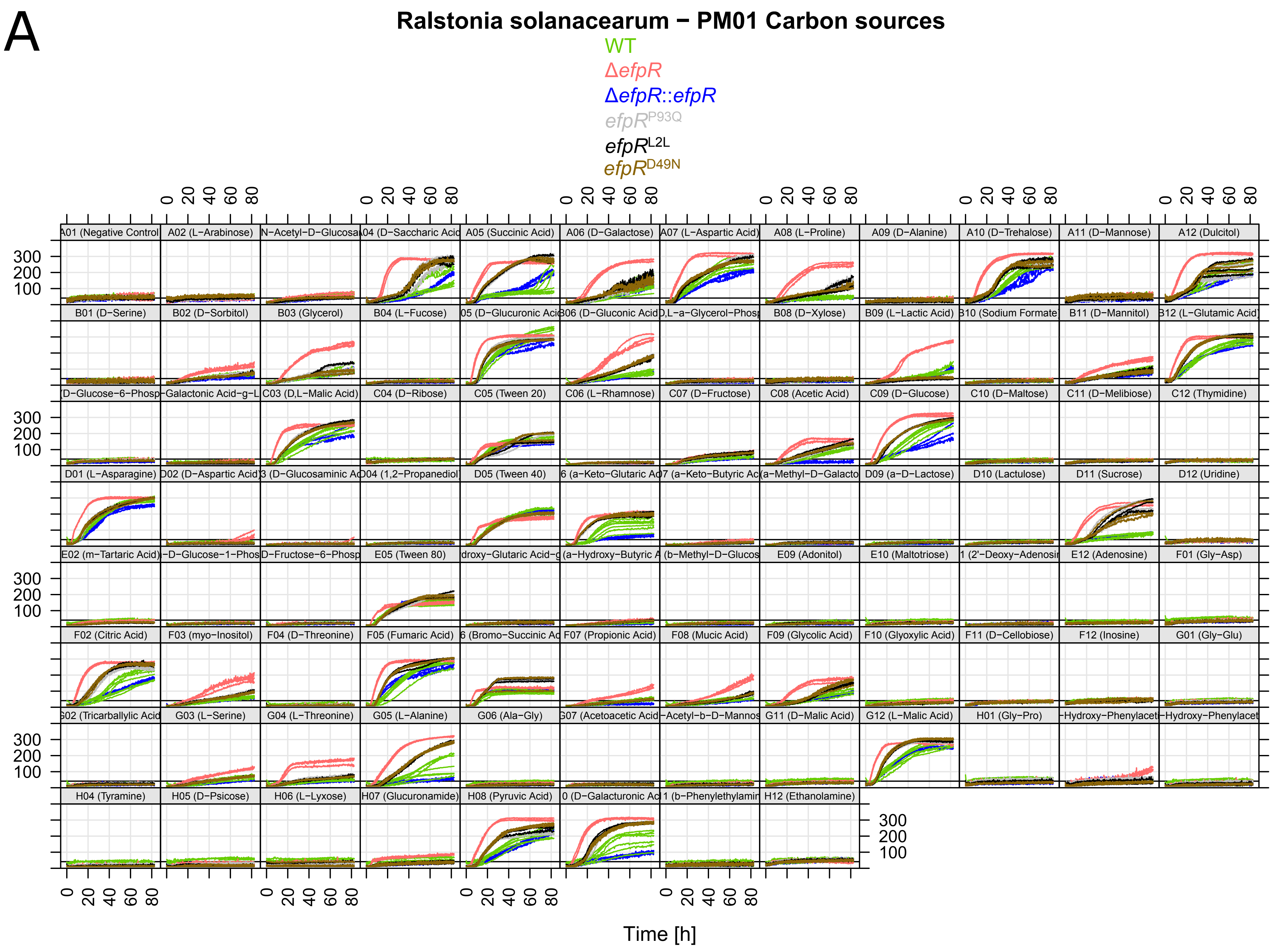
A
Ralstonia solanacearum − PM01 Carbon sources
WT
ΔefpR
ΔefpR::efpR
efpR P93Q
efpR L2L
efpR D49N
Value [OmniLog units]
Time [h]
A01 (Negative Control)
A02 (L−Arabinose)
A04 (D−Saccharic Acid)
A05 (Succinic Acid)
A06 (D−Galactose)
A07 (L−Aspartic Acid)
A08 (L−Proline)
A09 (D−Alanine)
A10 (D−Trehalose)
A11 (D−Mannose)
A12 (Dulcitol)
B01 (D−Serine)
B02 (D−Sorbitol)
B03 (Glycerol)
B04 (L−Fucose)
B08 (D−Xylose)
B09 (L−Lactic Acid)
B11 (D−Mannitol)
C03 (D,L−Malic Acid)
C04 (D−Ribose)
C05 (Tween 20)
C06 (L−Rhamnose)
C07 (D−Fructose)
C08 (Acetic Acid)
C09 (D−Glucose)
C10 (D−Maltose)
C11 (D−Melibiose)
C12 (Thymidine)
D01 (L−Asparagine)
D05 (Tween 40)
D09 (a−D−Lactose)
D10 (Lactulose)
D11 (Sucrose)
D12 (Uridine)
E05 (Tween 80)
E09 (Adonitol)
E10 (Maltotriose)
E12 (Adenosine)
F01 (Gly−Asp)
F02 (Citric Acid)
F03 (myo−Inositol)
F04 (D−Threonine)
F05 (Fumaric Acid)
F07 (Propionic Acid)
F08 (Mucic Acid)
F09 (Glycolic Acid)
F10 (Glyoxylic Acid)
F11 (D−Cellobiose)
F12 (Inosine)
G01 (Gly−Glu)
G03 (L−Serine)
G04 (L−Threonine)
G05 (L−Alanine)
G06 (Ala−Gly)
G11 (D−Malic Acid)
G12 (L−Malic Acid)
H01 (Gly−Pro)
H04 (Tyramine)
H05 (D−Psicose)
H06 (L−Lyxose)
H07 (Glucuronamide)
H08 (Pyruvic Acid)
H12 (Ethanolamine)
0 20 40 60 80
100 200 300

B

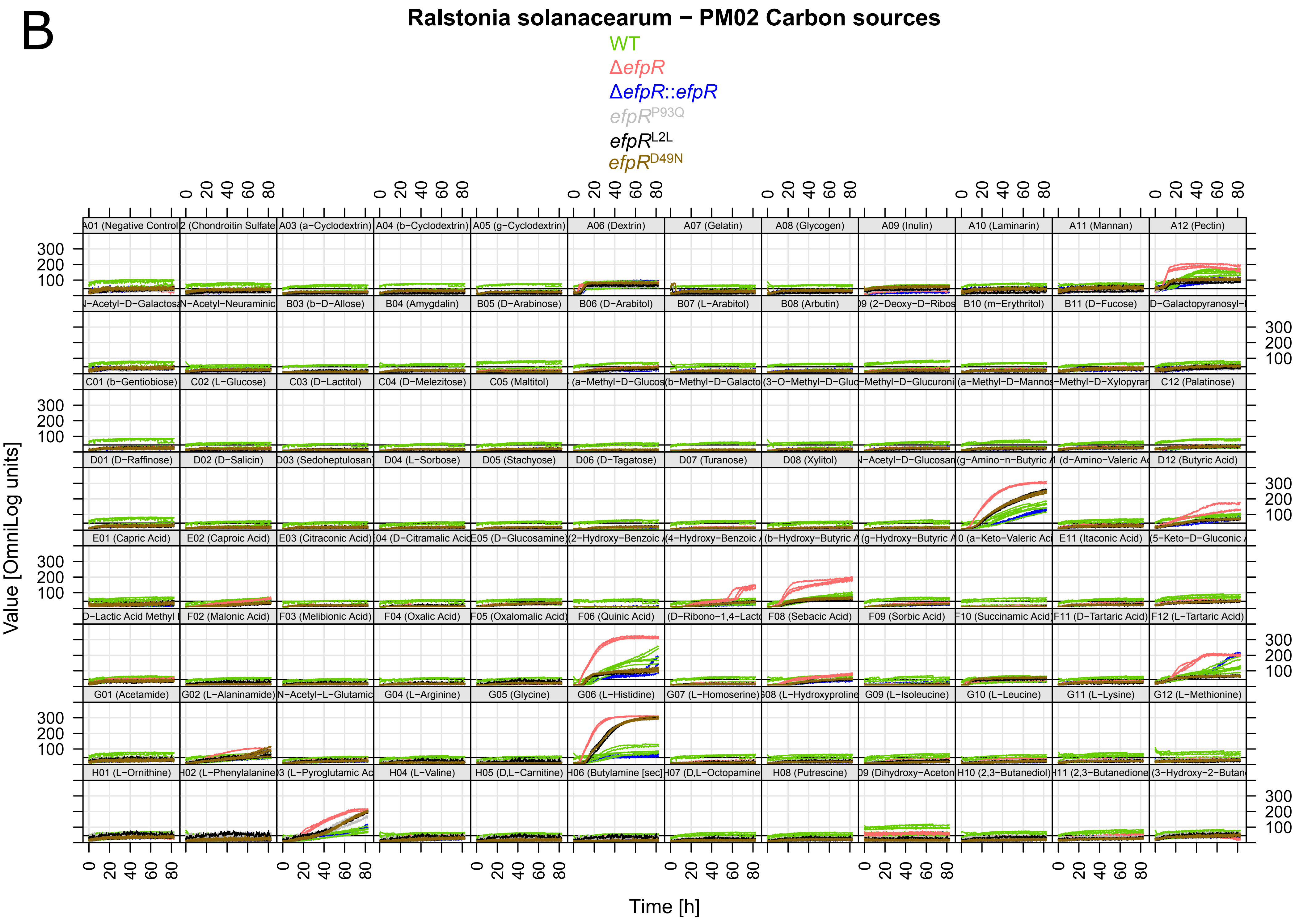

Ralstonia solanacearum − PM02 Carbon sources
WT
ΔefpR
ΔefpR::efpR
efpR P93Q
efpR L2L
efpR D49N
Value [OmniLog units]
Time [h]

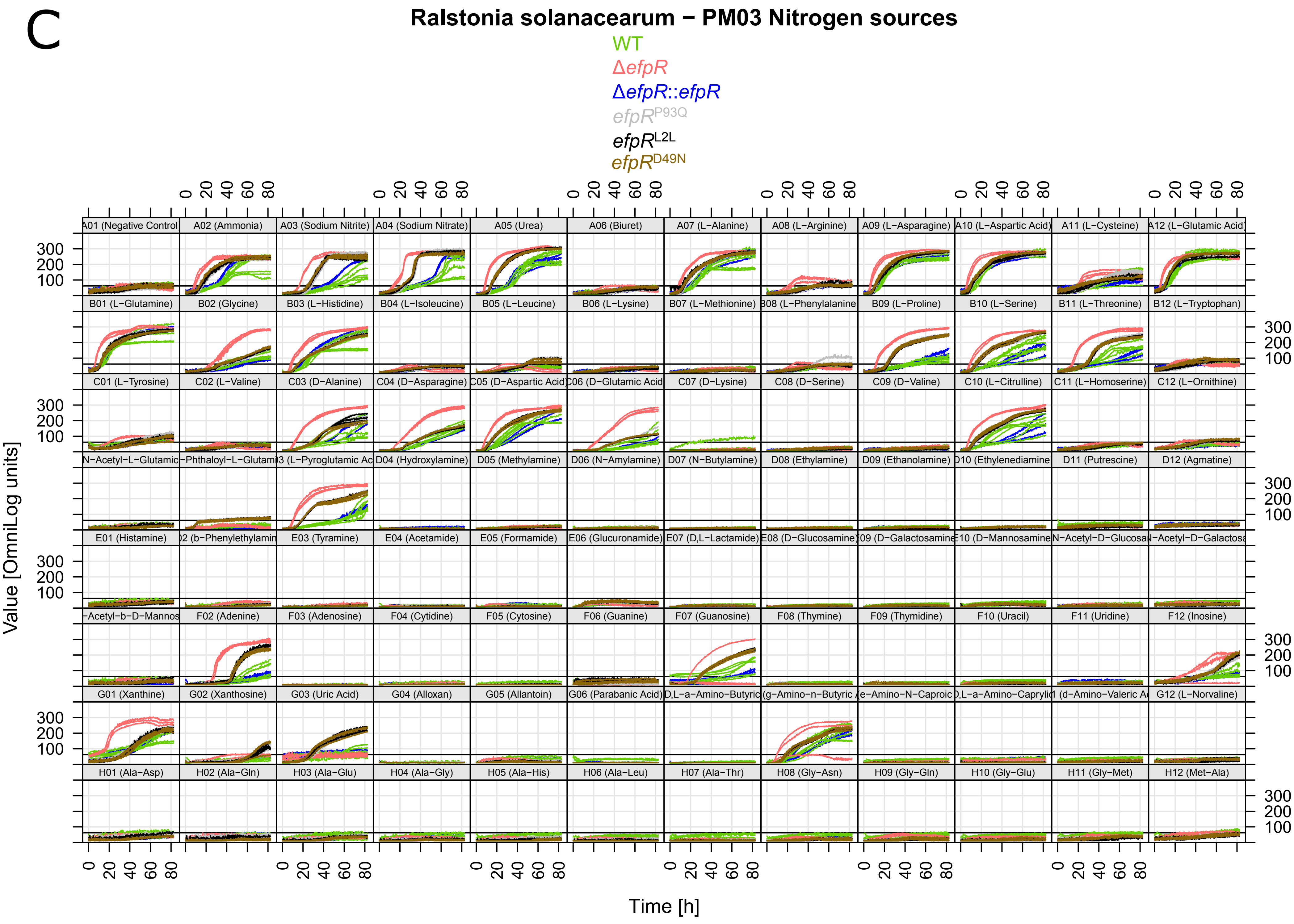
C
Ralstonia solanacearum − PM03 Nitrogen sources
WT
ΔefpR
ΔefpR::efpR
efpR P93Q
efpR L2L
efpR D49N
A01 (Negative Control
A02 (Ammonia)
A03 (Sodium Nitrite)
A04 (Sodium Nitrate)
A05 (Urea)
A06 (Biuret)
A07 (L−Alanine)
A08 (L−Arginine)
A09 (L−Asparagine)
A10 (L−Aspartic Acid)
A11 (L−Cysteine)
A12 (L−Glutamic Acid)
B01 (L−Glutamine)
B02 (Glycine)
B03 (L−Histidine)
B04 (L−Isoleucine)
B05 (L−Leucine)
B06 (L−Lysine)
B07 (L−Methionine)
08 (L−Phenylalanine
B09 (L−Proline)
B10 (L−Serine)
B11 (L−Threonine)
B12 (L−Tryptophan)
C01 (L−Tyrosine)
C02 (L−Valine)
C03 (D−Alanine)
C04 (D−Asparagine)
C05 (D−Aspartic Acid
06 (D−Glutamic Acid
C07 (D−Lysine)
C08 (D−Serine)
C09 (D−Valine)
C10 (L−Citrulline)
C11 (L−Homoserine)
C12 (L−Ornithine)
N−Acetyl−L−Glutamic
−Phthaloyl−L−Glutam
3 (L−Pyroglutamic Ac
D04 (Hydroxylamine)
D05 (Methylamine)
D06 (N−Amylamine)
D07 (N−Butylamine)
D08 (Ethylamine)
D09 (Ethanolamine)
10 (Ethylenediamine
D11 (Putrescine)
D12 (Agmatine)
E01 (Histamine)
2 (b−Phenylethylamin
E03 (Tyramine)
E04 (Acetamide)
E05 (Formamide)
E06 (Glucuronamide)
E07 (D,L−Lactamide)
E08 (D−Glucosamine)
09 (D−Galactosamine
10 (D−Mannosamine
N−Acetyl−D−Glucosa
−Acetyl−D−Galactos
−Acetyl−b−D−Mannos
F02 (Adenine)
F03 (Adenosine)
F04 (Cytidine)
F05 (Cytosine)
F06 (Guanine)
F07 (Guanosine)
F08 (Thymine)
F09 (Thymidine)
F10 (Uracil)
F11 (Uridine)
F12 (Inosine)
G01 (Xanthine)
G02 (Xanthosine)
G03 (Uric Acid)
G04 (Alloxan)
G05 (Allantoin)
G06 (Parabanic Acid)
D,L−a−Amino−Butyric
(g−Amino−n−Butyric
e−Amino−N−Caproic
D,L−a−Amino−Caprylic
(d−Amino−Valeric Ac
G12 (L−Norvaline)
H01 (Ala−Asp)
H02 (Ala−Gln)
H03 (Ala−Glu)
H04 (Ala−Gly)
H05 (Ala−His)
H06 (Ala−Leu)
H07 (Ala−Thr)
H08 (Gly−Asn)
H09 (Gly−Gln)
H10 (Gly−Glu)
H11 (Gly−Met)
H12 (Met−Ala)
Value [OmniLog units]
100
200
300
Time [h]
0
20
40
60
80
