# Supplementary material for: Enhanced in planta Fitness through Adaptive Mutations in EfpR, a Dual Regulator of Virulence and Metabolic Functions in the Plant Pathogen Ralstonia solanacearum
Source: PLoS Pathog. 2016 Dec 2;12(12):e1006044. doi: 10.1371/journal.ppat.1006044 (PMC5135139; doi:10.1371/journal.ppat.1006044)
Supplement: S5 Table — (PPTX) [file ppat.1006044.s010.pptx]

## Slide 1
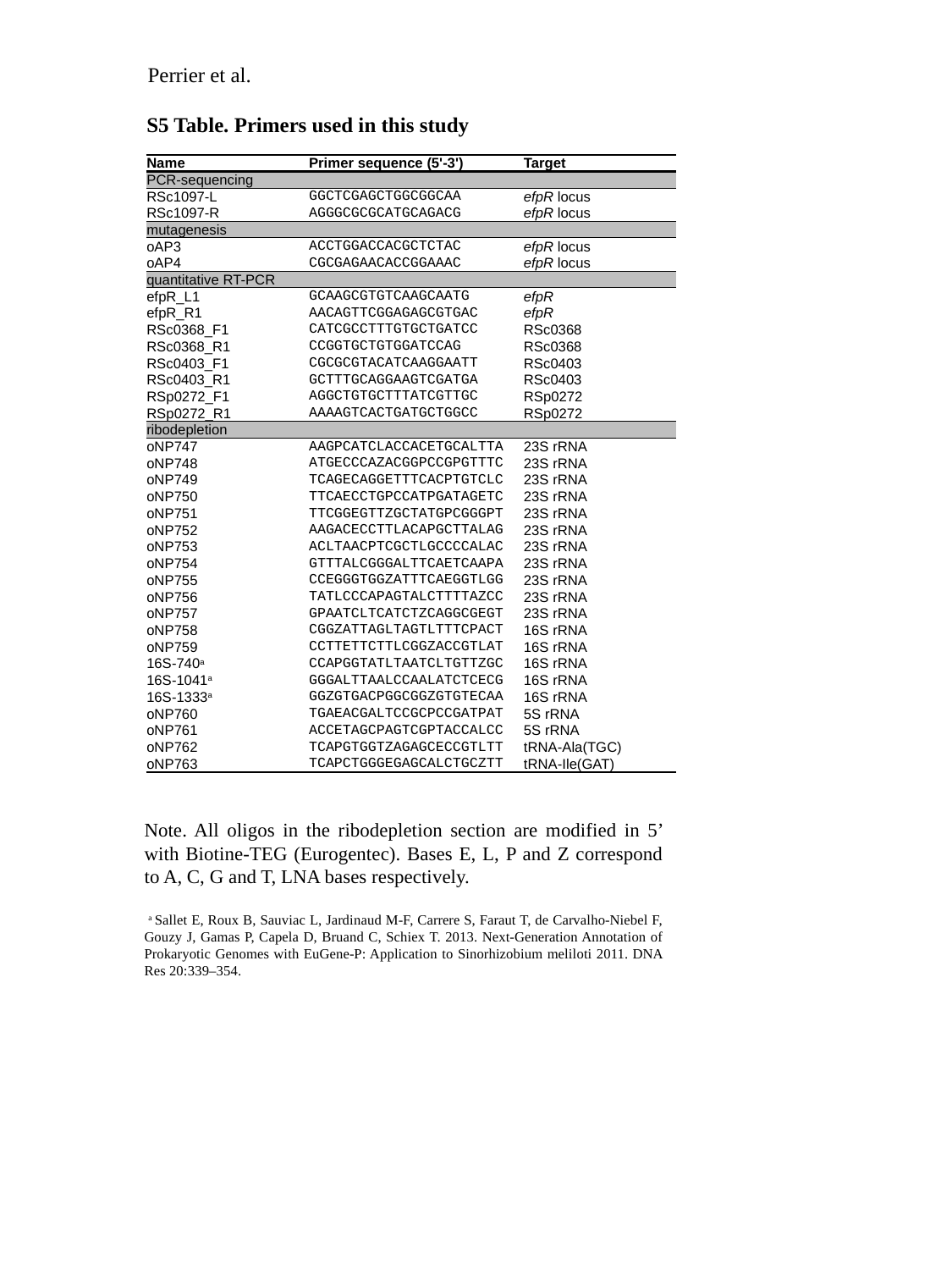

Perrier et al.
S5 Table. Primers used in this study
| Name | Primer sequence (5'-3') | Target |
| --- | --- | --- |
| PCR-sequencing | | |
| RSc1097-L | GGCTCGAGCTGGCGGCAA | efpR locus |
| RSc1097-R | AGGGCGCGCATGCAGACG | efpR locus |
| mutagenesis | | |
| oAP3 | ACCTGGACCACGCTCTAC | efpR locus |
| oAP4 | CGCGAGAACACCGGAAAC | efpR locus |
| quantitative RT-PCR | | |
| efpR\_L1 | GCAAGCGTGTCAAGCAATG | efpR |
| efpR\_R1 | AACAGTTCGGAGAGCGTGAC | efpR |
| RSc0368\_F1 | CATCGCCTTTGTGCTGATCC | RSc0368 |
| RSc0368\_R1 | CCGGTGCTGTGGATCCAG | RSc0368 |
| RSc0403\_F1 | CGCGCGTACATCAAGGAATT | RSc0403 |
| RSc0403\_R1 | GCTTTGCAGGAAGTCGATGA | RSc0403 |
| RSp0272\_F1 | AGGCTGTGCTTTATCGTTGC | RSp0272 |
| RSp0272\_R1 | AAAAGTCACTGATGCTGGCC | RSp0272 |
| ribodepletion | | |
| oNP747 | AAGPCATCLACCACETGCALTTA | 23S rRNA |
| oNP748 | ATGECCCAZACGGPCCGPGTTTC | 23S rRNA |
| oNP749 | TCAGECAGGETTTCACPTGTCLC | 23S rRNA |
| oNP750 | TTCAECCTGPCCATPGATAGETC | 23S rRNA |
| oNP751 | TTCGGEGTTZGCTATGPCGGGPT | 23S rRNA |
| oNP752 | AAGACECCTTLACAPGCTTALAG | 23S rRNA |
| oNP753 | ACLTAACPTCGCTLGCCCCALAC | 23S rRNA |
| oNP754 | GTTTALCGGGALTTCAETCAAPA | 23S rRNA |
| oNP755 | CCEGGGTGGZATTTCAEGGTLGG | 23S rRNA |
| oNP756 | TATLCCCAPAGTALCTTTTAZCC | 23S rRNA |
| oNP757 | GPAATCLTCATCTZCAGGCGEGT | 23S rRNA |
| oNP758 | CGGZATTAGLTAGTLTTTCPACT | 16S rRNA |
| oNP759 | CCTTETTCTTLCGGZACCGTLAT | 16S rRNA |
| 16S-740a | CCAPGGTATLTAATCLTGTTZGC | 16S rRNA |
| 16S-1041a | GGGALTTAALCCAALATCTCECG | 16S rRNA |
| 16S-1333a | GGZGTGACPGGCGGZGTGTECAA | 16S rRNA |
| oNP760 | TGAEACGALTCCGCPCCGATPAT | 5S rRNA |
| oNP761 | ACCETAGCPAGTCGPTACCALCC | 5S rRNA |
| oNP762 | TCAPGTGGTZAGAGCECCGTLTT | tRNA-Ala(TGC) |
| oNP763 | TCAPCTGGGEGAGCALCTGCZTT | tRNA-Ile(GAT) |
Note. All oligos in the ribodepletion section are modified in 5’ with Biotine-TEG (Eurogentec). Bases E, L, P and Z correspond to A, C, G and T, LNA bases respectively.
 a Sallet E, Roux B, Sauviac L, Jardinaud M-F, Carrere S, Faraut T, de Carvalho-Niebel F, Gouzy J, Gamas P, Capela D, Bruand C, Schiex T. 2013. Next-Generation Annotation of Prokaryotic Genomes with EuGene-P: Application to Sinorhizobium meliloti 2011. DNA Res 20:339–354.
